# Measures of Facilitator Competent Adherence Used in Parenting Programs and Their Psychometric Properties: A Systematic Review

**DOI:** 10.1007/s10567-021-00350-8

**Published:** 2021-05-21

**Authors:** Mackenzie Martin, Bridget Steele, Jamie M. Lachman, Frances Gardner

**Affiliations:** Centre for Evidence-Based Intervention, Department of Social Policy and Intervention, University of Oxford, Oxford, UK

**Keywords:** Parenting, Facilitator, Implementation science, Psychometrics, Systematic review

## Abstract

Implementation fidelity is a critical component of intervention science, which aims to understand how interventions unfold in practice to improve outcomes. A key element of fidelity is facilitator competent adherence—the extent to which a program is delivered as prescribed with the specified level of quality. We conducted a two-part systematic review examining these aspects in parenting programs aiming to reduce child behavior problems and maltreatment. Part One reviews measures of facilitator competent adherence and Part Two examines the psychometric properties of the observational measures found. Searches identified 9153 articles from electronic databases, citation tracking, and expert input. After screening using pre-specified criteria, 156 (Part One) and 41 (Part Two) articles remained. In Part One, measure, facilitator, and intervention characteristics were extracted and synthesized from 65 measures. Most measures were observational, used by facilitators and researchers, and employed Likert-scale ratings. In Part Two, evidence on the reliability (internal consistency, inter-rater, intra-rater, test–retest) and validity (content, construct, convergent/divergent, criterion) of 30 observational measures identified from Part One was synthesized and evaluated. An adapted COSMIN checklist was used to assess study and measure quality. We found most studies to be of reasonably high quality. This is the first review to summarize and critically appraise measures of facilitator competent adherence used in the parenting program literature and establish their psychometric properties. The findings underscore the need to advance research on measures of facilitator competent adherence; reliable, valid, and high-quality implementation measures allow for evidence-based decisions regarding the delivery and scale-up of parenting programs. PROSPERO Registration Number: CRD42020167872.

**Supplementary Information:**

The online version contains supplementary material available at 10.1007/s10567-021-00350-8.

## Introduction

Implementation science is a critical component of intervention research and the evidence-based movement (Fixsen et al., 2019). As is often commented upon in the intervention literature, there is a ‘science to service gap,’ (Fixsen et al., 2009) with many evidence-based programs not implemented beyond initial effectiveness studies. Implementation science seeks to fill this gap by examining how interventions actually unfold in practice and using this information to improve interventions, their implementation, and outcomes (e.g., Bhattacharyya et al., 2009; Mihalic, 2004; Peters et al., 2013). One way to evaluate the extent to which programs are delivered in practice is by measuring implementation fidelity (Proctor et al., 2011).

This paper focuses on two related aspects of implementation fidelity—facilitator adherence and quality of delivery. These aspects were chosen as facilitators—the practitioners or lay people who implement programs (Fixsen et al., 2005)—are the vehicle through which participants receive an intervention (Petersilia, 1990). Facilitator adherence, or fidelity, is the strictness with which a facilitator implements the prescribed intervention content, activities, and strategies whereas facilitator quality of delivery, or competence, refers to the skill and style with which a facilitator delivers program components in practice (Dane & Schneider, 1998; Dusenbury et al., 2003; Fixsen et al., 2005). The combination of adherence and competence, or facilitator competent adherence, is the skill with which a facilitator delivers intervention components and the strictness with which they implement the program manual (Forgatch et al., 2005). It is important to distinguish facilitator competent adherence from other types of adherence referred to in the parenting program literature. For instance, some studies report on the level with which parents adhere to program components, such as by documenting their attendance to and implementation of home activities (e.g., Nock & Ferriter, 2005).

The focus of this paper is facilitator competent adherence among facilitators of parenting programs aiming to (a) reduce child maltreatment, harsh or dysfunctional parenting, and/or child conduct problems and/or (b) improve positive child behavior management strategies, parent–child bonding attachment and relationships, and/or early child development outcomes. These programs have varying theoretical underpinnings, core components, session formats (e.g., group or one-on-one), and program faciltiators (e.g., lay or professionals). Parenting programs with these aims and varying approaches were chosen because there is a sizeable body of evidence on their effectiveness (e.g., Chen & Chan, 2016; Furlong et al., 2013). Knowledge of the competent adherence with which faciltiators deliver parenting programs may illuminate how to improve facilitator delivery and thereby how to enhance family outcomes.

### Measures of Competent Adherence

Numerous parenting programs use observational or non-observational measurement tools to assess facilitator competent adherence. Observational methods require assessors to watch live or video-recorded sessions whereas non-observational methods require assessors to listen to audio recordings of sessions or use facilitator self-report (Girard & Cohn, 2016). Although time and resource intensive (Horvath et al., 2011), observational methods may be more accurate due to their objectivity and ability to capture facilitator and participant body language (Gardner, 2000). Non-observational methods are less reliable due to factors such as social desirability (e.g., facilitator self-reports) (Stone et al., 2000) and because they miss capturing important elements of delivery, including participation engagement (e.g., audio assessments). However, with observational methods, the presence of an observer or video-recorder may alter the facilitator’s natural delivery and, thus, introduce reactivity bias (Gardner, 2000).

Measures of facilitator competent adherence frequently use response types with dichotomous (e.g., ‘yes’ and ‘no’), frequency, or Likert-scale options. Dichotomous responses are clear cut, making it easier to establish inter-rater reliability between assessments, yet may miss nuances in program delivery, particularly intricacies associated with measuring skills. Frequency items ask assessors to capture the number of times a facilitator implements a particular activity or skill. With Likert items, assessors are given options that capture gradations in delivery that make it challenging to establish reliability.

Measures can also be designed to capture varying combinations of competence and adherence; measures may assess competence-only, adherence-only, competent adherence, or competence and adherence. Competence-only measures seek to assess the quality with which facilitators deliver a program. Competence is a subjective concept making the development of a tool to measure it complex; it is difficult to pinpoint, and therefore assess, the precise impactful outward manifestations of high-quality delivery (Mowbray et al., 2003). Using such a tool is challenging as interpretations of what is being observed may differ by assessor, thus varying their ratings on measure items. Adherence-only measures assess the extent to which facilitators implement a program as designed. Previous literature indicate that adherence-only measures are most common, potentially because they are quick and simple to design and use (Goense et al., 2015) but may miss capturing the nuance of program delivery. Measures of competent adherence detail whether and how well a facilitator delivers a program as intended. Measures that assess both domains are particularly insightful as they attempt to capture a complete picture of facilitator delivery.

### Study Justification and Objectives

It is important to study facilitator delivery as stronger facilitator delivery may be associated with better program outcomes. While some research in the parenting intervention literature has found that higher facilitator competent adherence is associated with better intervention outcomes (e.g., Eames et al., 2009, 2010; Forgatch et al., 2011; Hogue et al., 2008; Huey et al., 2000), other research has found the relationship unclear (e.g., Breitenstein et al., 2010; Cantu et al., 2010). The literature on the association between fidelity and outcomes may be mixed for a variety of reasons, including the difficulty of its study, the potential influence of confounding variables, and inaccurate measurement (such as by using measures that are not reliable and/or valid). Although numerous parenting programs have assessed facilitator competent adherence, no studies have systematically reviewed the measures used in these studies or documented their psychometric properties. Given measures of competent adherence capture a variety of dimensions using a range of approaches, this review provides a summary of their characteristics. The resulting synthesis is intended to provide practitioners and researchers with fundamental information and insights to assist them in selecting, adapting, developing, and using measures for their programs and studies. Even though many measures exist, they may not be reliable and/or valid. It is fundamental that fidelity measures are reliable and valid as these properties speak to whether they can be administered consistently and capture what they intend to measure (Mowbray et al., 2003). Information on measure reliability and validity is beneficial as it provides practitioners and researchers with a picture of the quality of implementation measures currently available; supports researchers in determining which measures need further testing and analysis; and it allows practitioners and researchers to quickly assess the desirability of using measures in future program implementation and evaluation. Thus, in compiling and analyzing the characteristics of measures of facilitator competent adherence used in parenting programs and by evaluating the psychometric properties and practicality of the observational measures found, this review takes the first step towards a comprehensive understanding of the role facilitator delivery plays in program effectiveness.

## Methods

The review has two parts. Part One identifies and synthesizes data about the measures of facilitator competent adherence used in the parenting program literature. Part Two reports on the quality and psychometric properties of the observational measures of facilitator competent adherence identified in Part One. The study was pre-registered on PROSPERO (CRD42020167872).

### Search Strategy

We developed a search string informed by related studies and systematic reviews (Barlow et al., 2017; Gardner et al., forthcoming):

(parent* OR caregiver* OR guardian* OR carer*.ab) AND (training OR program* OR intervention* OR treatment OR trial* or prevention.ab) AND (competen* OR quality OR adheren* OR fidelity* OR integrity OR compliance.ab) AND (child* OR kid* OR adolesc* OR teen* OR youth* OR baby OR babies OR toddler* OR neonate* OR infant* OR newborn OR juvenile* OR minor* OR early child* OR ECD.ab) AND (facilitator* OR practitioner* OR therapist* OR clinician* OR teacher* OR worker* OR provider* OR leader* OR specialist* OR professional* OR coordinator* OR administrator* OR counsellor* OR counselor* OR implementer* OR coach* OR instructor* OR trainer* OR mentor* OR educator*.ab) AND (scale* OR subscale* OR tool* OR measure* OR instrument* OR report* OR index* OR checklist* OR test*.ab).

We conducted our search in the following databases: Applied Social Sciences Index and Abstracts, Cochrane Database of Systematic Reviews, Cochrane Central Register of Controlled Trials (CENTRAL), EconLIT, PsycINFO, EBSCO combined search (CINAHL, ERIC, MEDLINE), Global Health, The International Bibliography of the Social Sciences (IBSS), Social Science Premium Collection, and ProQuest Dissertations and Theses. Articles published from inception until July 2020 were eligible for inclusion. The initial search was conducted in January of 2020 and updated in July 2020.

We hand searched articles included in Gardner et al.’s (forthcoming) review to ensure representation of parenting programs from low- and middle-income countries (LMICs), where there are typically fewer studies. Backward reference searching was conducted using the reference lists of all included articles, and forward reference searching was conducted using Google Scholar. We also asked experts focused on parenting program fidelity, including Bearrs, Eames, Forgatch, Hogue, and Smith, to share relevant published or ongoing studies. Finally, once a list of measures used in each included study was generated, we searched the names of these measures in the databases listed above to capture any additional articles.

### Study Selection, Data Extraction, and Analysis

#### Part One

Articles were screened using pre-specified criteria regarding the type of programs, measures, and articles eligible for inclusion. Articles on parenting programs aiming to (a) reduce child maltreatment, harsh, or dysfunctional parenting, and/or child conduct problems and/or (b) improve positive child behavior management strategies, parent–child bonding/attachment and relationships, and/or early childhood development outcomes were included. However, programs which (1) narrowly focused on specific child risks such as poisoning or accidents or on skills training for children’s specific medical conditions or physical disabilities (e.g., developmental disability) or (2) primarily delivered financial support (e.g., conditional cash transfer programs) or other support to parents, but did not aim to change parents’ knowledge or behavior concerning their child(ren), were not included. To be included, at least 50% of program content needed to be delivered to parents/caregivers. Further, parents needed to be at least 18 years old and children needed to be 17 years of age or younger. To be included, measures needed to report on facilitator competent adherence and provide some reference to how the measure was used. Measures reporting solely on other implementation fidelity dimensions such as treatment alliance were not included. Academic or gray publications (peer-reviewed articles, unpublished manuscripts, ongoing studies, and theses/dissertations) reporting on programs in any geographic region were included provided that the publications were in English.

Data on measure, facilitator, and intervention characteristics were extracted from included studies. The measure characteristics extracted were name of the measure, domain(s) of adherence and/or competence measured, other fidelity domain(s) captured, number of items, types of response options, type of assessors, mode of data collection, measure format, number of subscales, length of assessment, cost of assessment, accessibility of the measure, assessor training, length of assessor training, and measure result(s). The facilitator characteristics extracted were age, sample size, gender, educational background, years of career experience, parenting program experience, and other relevant information. The intervention characteristics extracted were the program title/brand, objectives, primary/secondary outcomes, age of parents/caregivers, age of children, country of study, and other relevant information. We narratively synthesized and compiled the data in tables to provide an overview of and identify gaps in the literature reviewed.

#### Part Two

Articles included in Part One were included in Part Two if they reported on an observational measure as these provide more complete and detailed accounts of program delivery (Eames et al., 2008). Additionally, studies needed to report on at least one of internal consistency, inter-rater reliability, intra-rater reliability, test–retest reliability, content validity, construct validity, convergent validity, divergent validity, or criterion validity. At last, a study that only examined inter-rater reliability needed to report on the reliability of assessors using observational measures. Studies in which inter-rater reliability was established between assessor and facilitator self-reports were excluded as these examined the reliability of self-reports.

The COSMIN initiative recommendations guided our data extraction and analysis on evaluating psychometric properties and conducting systematic reviews of measures (Prinsen et al., 2018). We analyzed and reported on how many studies documented each property, the evidence for each property, the range of statistics provided, and the strength of the evidence.

#### Reliability Data

The review examined four types of reliability—internal consistency, intra-rater reliability, inter-rater reliability, and test–retest reliability. Internal consistency (consistency among measure items) as reported by Cronbach alphas, Person separation indices, Spearman and Pearson correlations, and intra-class correlations (ICCs) was extracted, wherein high correlations generally indicate stronger consistency (Terwee et al., 2007). Inter-rater (consistency between assessors), intra-rater (consistency of an assessor), and test–retest reliability (consistency of facilitator delivery) were extracted. Percentage agreements and Cohen’s Kappas were collected to assess how often assessors rate measure items exactly the same way and ICCs, correlations, and Cronbach Alphas were collected to determine the extent to which assessors generally rate items the same way (Stemler & Tsai, 2008).

#### Validity Data

The review evaluated four types of validity—content, construct, convergent/divergent, and criterion—to capture the extent to which a measure actually assesses competent adherence (Mokkink et al., 2010a). Content validity information was extracted regarding how a measure was developed and assessed so as to determine whether key stakeholders judge the measure as capturing competent adherence and whether the measure is meaningful for use in practice (Terwee et al., 2018). To assess the extent to which measures statistically reflect their intended underlying concepts, construct (or structural) validity information was extracted regarding the type of factor analysis used, number of factors that emerged, factor loadings, percentage of variance explained by the factors, eigenvalues, and model fit (e.g., model chi square, confirmatory factor index, root mean square of approximation, standardized root mean square of approximation) (Terwee et al., 2007). Information about convergent and divergent (or discriminant) validity as reported by correlations among measures was extracted to assess the degree to which a measure is statistically similar to or different from other measures designed to capture similar or different constructs (Terwee et al., 2007). Information about criterion validity as reported by correlations between measures was extracted to assess the extent to which a measure is correlated with a gold standard measure which has undergone rigorous psychometric testing and found to be of high quality (Swerdlik & Cohen, 2005).

### Risk of Bias and Quality Assessment

We evaluated the quality and risk of bias of both the included studies and measures identified using three risk of bias and quality checklists: one to evaluate measure properties, one to evaluate studies, and one to evaluate measure practicality. Each checklist is composed of questions outlining high- and low-quality criterion on a number of dimensions wherein ‘+’ represents high-quality, ‘−’ represents low-quality, and ‘?’ indicates insufficient information.

A Measure Risk of Bias and Quality Checklist was developed based on the COSMIN guidelines including adaptations made by other authors (Gridley et al., 2019; Mokkink et al., 2010a, 2010b; Terwee et al., 2007). A three-item Study Risk of Bias and Quality Checklist was developed based on relevant literature. Item one addresses the session sampling method with high-quality studies either observing or rating all program sessions or randomly selecting sessions for observation to reduce selection bias (Ellenberg, 1994; Walton et al., 2017). Item two addresses assessor bias with high-quality studies using two or more independent assessors to conduct observations so that assessments are not biased and so that inter-rater reliability can be established (Hallgren, 2012; Walton et al., 2017). Item three considers the role of facilitator reactivity with high-quality studies providing information on steps taken to reduce reactivity (Gardner, 2000; Kazdin, 1982).

We also developed a three-item checklist to evaluate measure practicality based on the feasibility and sustainability of the training provided to assessors, the measure’s utility in practice, and the availability of the measure in-text or online (Barkham et al., 1998; Milne et al., 2011).

### Inter-coder Reliability

To ensure replicability, inter-coder reliability was established at the title/abstract screening, full-text screening, and data extraction stages (Belur et al., 2018). Following training, the second author independently coded a random selection of 10% of the articles at each stage of study inclusion (Lombard et al., 2017). Discrepancies were resolved through discussion.

## Results: Part One

### Search Results

**Fig. 1:**
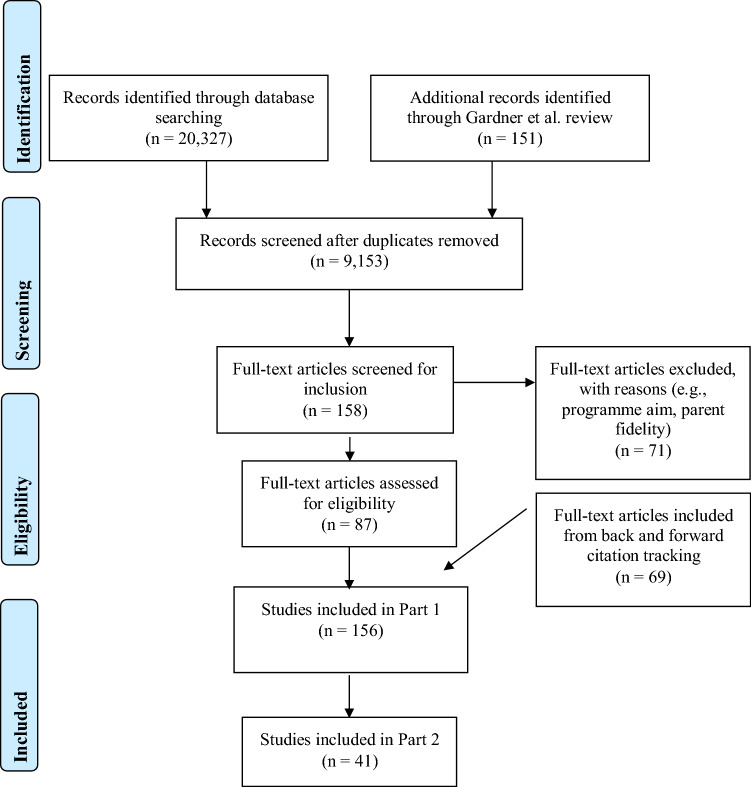
PRISMA flowchart of study screening and selection

Electronic bibliographic searches surfaced 20,327 articles. Further, Gardner et al. shared 151 articles included in their review. No new measures surfaced from the 20 experts contacted. After duplicate removal, 9153 articles remained. Article screening resulted in 156 studies included in Part One and 41 in Part Two. All searches were conducted on the same day in January 2020 and updated in July 2020 (Fig. 1).

### Inter-rater Reliability of Review Coders

Reliability between coders was 94.4% at Part One title/abstract screening, 100% at Part One full-text screening, 92.8% at Part One data extraction 1, 93.8% at Part Two full-text screening, and 100.0% at Part Two data extraction.

### Description of Measures

Table 1 describes the characteristics of the measures found in the included studies. The 156 Part One studies assessed 65 measures of which 46 were named and 19 were unnamed. The most commonly reported were Therapy Adherence Measure (*n* = 16); Fidelity of Implementation Rating Scale (*n* = 8); Therapy Adherence Measure-Revised (*n* = 7); COACH Rating System (*n* = 5); and the Leadership Observation Tool (*n* = 3).

**Table 1:** A summary of the part one measure characteristics results

Domain	Number (%) of studies reporting each domain (*N* = 151)	Assessor type	Number (%) of studies reporting each assessor type (*N* = 135)	Response option	Number (%) of studies reporting each response option (*N* = 107)	Mode of data collection	Number (%) of studies reporting each mode of data collection (*N* = 133)
Adherence only	73 (49.7%)	Facilitators only	21 (15.6%)	Dichotomous only	11 (10.3%)	Observational	53 (40.0%)
Competence only	4 (2.6%)	Researchers only	19 (14.1%)	Likert only	73 (68.2%)	Non-observational	55 (41.4%)
Competent adherence	52 (34.4%)	Third party only	17 (12.6%)	Dichotomous and Likert	8 (7.5%)	Both observational and non-observational	25 (18.8%)
Competence and adherence separately	21 (13.9%)	Parents only	12 (8.9%)	Dichotomous and other	0 (0.0%)		
Competent adherence and adherence	1 (0.7%)	Supervisors only	10 (7.4%)	Likert and other	14 (13.1%)		
		Combination	29 (21.5%)				
		Other	12 (8.9%)	Other (minutes, frequency, symbols) only	1 (0.9%)		
		Combination and other	15 (11.3%)				

Of the 151 studies reporting on domains measured, 73 articles (48.3%) captured adherence-only, 52 (34.4%) captured competent adherence, 21 (13.9%) captured competence and adherence, four (2.6%) captured competence-only, and one (0.7%) captured adherence and competent adherence.

One hundred and thirty-three (85.3%) studies reported on the mode of data collection used to make assessments with 53 (40.0%) using observational methods, 55 (41.4%) using non-observational methods, and 25 (18.8%) using both. These approaches included video (31.6%), memory (29.3%), combination (21.8%), audio (6.8%), live (6.8%), and other (3.0%) (live or video; case notes).

One hundred and thirty-five (86.5%) studies reported on the type of individual who conducted assessments. Assessors were facilitators (15.6%), researchers (14.1%), third parties (12.6%), parents (8.9%), supervisors (7.4%), combinations of assessors (21.5%), ‘other’ (including program trainers, adolescents, community workers, independent facilitators, experts, and psychologists) (8.9%), or combinations and other (11.3%).

One hundred and seven (68.6%) studies reported on response options, which were dichotomous, Likert-scale, frequency, ‘other,’ or combinations of formats. Nineteen studies reported dichotomous formats either alone or in combination. Of these, 11 measured adherence-only, four measured competent adherence, four measured competence and adherence, and none measured competence-only. Thus, all studies using dichotomous measures reported on adherence either alone or in combination with competence. Ninety-five reported Likert response options either alone or in combination with other item formats, with most providing a definition (e.g., 5 = very much) for each Likert point. Response options ranged from 0 to 11. Of these 95 studies, 43 measured competent adherence, 20 measured competence and adherence, 30 measured adherence-only, and two measured competence-only. Of fifteen studies reporting frequency and ‘other’ formats (some in combination), five recorded minutes, eight coded the frequency of activities or skills, and two used a coding system with the notations ‘+’, ‘−’, and ‘not applicable’ to report on the assessment of facilitator delivery.

Fifty-two (33.3%) studies reported on whether assessors were provided with training on how to conduct assessments. Sixteen (30.8%) reported on the amount of training received, which averaged 20 h. Twenty-eight (53.8%) reported providing training for assessors measuring competent adherence, nine (17.3%) for competence and adherence, 15 (28.8%) for adherence-only, and none for competence-only. Thirty-two (61.5%) reported training for assessors of observational measures, 12 (23.1%) for non-observational measures, seven (13.5%) for both methods, and one (1.9%) did not report on the mode(s) of data collection. All training was provided to third-party assessors (e.g., researchers, program staff) and none was provided to facilitator or parent assessors. One study reported how long it took to complete an assessment. No studies reported on the cost of training assessors and conducting assessments.

### Description of Facilitators

The results regarding facilitator characteristics are described in Table 2. Thirty-eight (24.4%) studies reported on facilitator age which ranged from 23 to 68 years, with most studies (89.5%) having some or all facilitators between 31 and 40 years. Ninety-seven (62.2%) studies reported on the sample size of facilitators studied. The average sample was 65.8 with a median of 20.0 facilitators.

**Table 2:** A summary of the part one facilitator characteristics results

Facilitator education category	Number of studies reporting each facilitator education category (*N* = 90 total)	Facilitator years of experience or times delivering a parenting program	Number of studies reporting facilitator years of experience or times delivering a parenting program (*N* = 28 total)	Facilitator sample size	Number of studies reporting facilitator sample size (*N* = 97 total)	Facilitator years of career work experience	Number of studies reporting facilitator years of career work experience (*N* = 23 total)	Facilitator ethnicity	Number of studies reporting facilitator ethnicity (*N* = 41)	Facilitator age	Number of studies reporting facilitator age (*N* = 38 total)
No relevant formal education	6	No experience	2	1–10	28	None	0	Caucasian	33	20–30	21
Undergraduate degree	29	1–2 years	10	11–20	22	1–2 years	16	African American	17	31–40	34
Master’s degree	47	3–4 years	8	21–30	10	3–4 years	20	Hispanic or Latino	17	41–50	26
Doctoral degree	34	5–6 years	10	31–40	4	5–6 years	23	Asian or Pacific Islander	8	51–60	22
Psychology, counselling, or psychotherapy	27	7–8 years	5	41–50	3	7–8 years	17	Indigenous	4	61–70	6
Social work	28	9–10 years	5	51–60	3	9–10 years	15	‘Mixed,’ ‘Other,’ ‘Not Described’	5		
Teaching or education	8	1–2 times	3	61–70	5	11–20 years	16				
Public health	4	3–4 times	4	71–80	3	21–30 years	7				
Marital or family therapy	9	5–6 times	2	81–90	0	31 + years	5				
Nursing	6	7–8 times	1	91–100	1						
Mental health	6	9–10 times	3	101 +	18						
Childcare work	1	11 + times	3								
Human relations	1										
Faith-based work	3										
Child development	1										
Policy	1										
Applied behavioral analysis	1										
Child welfare	1										
Occupational or speech therapy	1										
Other and unspecified	9										

Please note that because articles reported having facilitators in many of the categories above, the numbers in the columns are not supposed to add up to the numbers set out in the headings

Fifty-seven (36.5%) studies reported on facilitator gender. The average percentage of female and male facilitators was 77.5% and 22.5%, respectively. Seven (12.3%) studies reported all-female teams. Twenty-eight (17.9%) studies reported on the amount of experience (years or number of times) facilitators had delivering parenting programs.

Ninety (57.7%) studies reported on the educational background of facilitators. Of these, 47 reported facilitators with a master’s degree (52.2%), 34 with a doctorate (37.8%), and 29 with an undergraduate degree (32.2%). Backgrounds in psychology (27 studies) and social work (28 studies) were most common. Mean years of career experience were 7.5 years in the 23 (67.6%) studies providing this information. Forty-one studies (26.3%) documented facilitator ethnicity.

### Description of Interventions

The 156 studies reported on 63 different parenting programs, with some reported in more than one study. The programs reported most frequently were MultiSystemic Therapy (*n* = 22), Incredible Years (*n* = 16), Parent–Child Interaction Therapy (*n* = 8), Parent Management Training—Oregon Model (*n* = 8), and Triple P (*n* = 6). The studies sought to address one or more of the parent and child outcomes of interest, including child behavior (*n* = 119), positive parent–child relationships and interactions (*n* = 29), child maltreatment (*n* = 21), behavior management (*n* = 17), early childhood development (*n* = 13), parent–child attachment and bonding (*n* = 10), and harsh or dysfunctional parenting (*n* = 6). The studies were conducted in 35 countries. Ninety-eight (62.3%) studies were conducted in the USA, 23 (14.7%) in England, Wales, or Ireland, and seven (4.5%) in the Netherlands. Only eight (5.1%) were conducted in LMICs. Thirty-eight (24.4%) studies reported on parent/caregiver ages, which ranged from 20.0 to 75.5 years with a mean of 39.9 years. One hundred and eleven studies (71.2%) reported on child age with 12 reporting on infants (0–1 year), 24 on toddlers (1–3), 47 on preschoolers (3–5), 57 on middle childhood (6–11), 61 on young adolescents (12–14), and 44 on older adolescents (15–17).

## Results: Part Two

The studies included in Part Two reported on 13 named and 17 unnamed observational measures in 22 different programs (Tables 3 and 4). Of the named measures, eight (61.5%) aimed to capture competent adherence, three (23.1%) focused on competence, and two (15.4%) focused on adherence. All but one used video methods alone or in combination. The studies reported using a multiplicity of assessor types, including independent or third-parties (*n* = 15), researchers (*n* = 13), supervisors (*n* = 4), independent program facilitators (*n* = 3), researchers and program specialists (*n* = 2), program specialists/trainers (*n* = 1), and unspecified (*n* = 4).

**Table 3:** A list of the parenting programs from studies in part two

Parenting program	Number of studies reporting on the program
Incredible years	7
Family check-up	6
Parent management training—oregon model (PMTO)	6
Multi-dimensional family therapy	5
Early head start	2
Parent child interaction therapy	2
Strengthening families program	2
Attachment and behavioural catch-up	1
Comet	1
Common sense parenting	1
Connect	1
Cope	1
Early intensive behaviour intervention	1
Familias Unidas	1
Multi-dimensional family prevention	1
New beginnings program	1
Parent–Child Care (PC-CARE)	1
Parenting with love and limits	1
Play and language for autistic youngsters (PLAY)	1
Sinovuyo teen (parenting for lifelong health for adolescents)	1
Strong African American families program	1
Triple P	1

**Table 4:** A summary of measures from included studies in part two

Names of the measures	Domain	Live, video, or both	Number of studies
No name	Various	Various	17
Fidelity of implementation rating system (e.g., Forgatch et al., 2005)	Competent adherence	Video	6
COACH rating system (e.g., Smith et al., 2013)	Competent adherence	Video	5
Home visitation observation form (e.g., Roggman et al., 2001)	Competent adherence	Video	2
Leadership observation tool (Eames et al., 2007, 2009)	Competent adherence	Video	2
Feedback observer global ratings form (Bustos, 2011)	Competence	Video	1
FIRST coach coding system (Snider, 2019)	Competence	Video	1
Parent program implementation checklist (Bywater et al., 2019)	Competent adherence	Live or video	1
Therapist Behaviour Rating Scale—2nd Version (Hogue et al., 2005; Singer, 2001)	Competent adherence	Video	2
Therapist Behaviour Rating Scale—Competence (Hogue et al., 2008)	Competence	Video	1
Therapist Behaviour Rating Scale (Hogue et al., 1998)	Competent adherence	Video	1
Therapist Skill Scale (Scott et al., 2008)	Competent adherence	Video	1
Treatment Integrity Checklist (Snider et al., 2019)	Adherence	Video	1
Video Supervision Manual (Sterrett-Hong et al., 2017)	Adherence	Video	1

### Study Risk of Bias and Quality

Each study was evaluated using the Study Risk of Bias and Quality Checklist (Table 5).

**Table 5:** A summary of the study risk of bias and quality checklist and measure practicality checklist results

Study	Study risk of bias and quality checklist			Measure practicality checklist		
	Sampling	Assessors	Reactivity	Feasibility and sustainability of training	Utility	Availability
Askeland et al. (2019)	−	+	?	+	+	−
Berkel et al. (2018	−	+	?	?	?	+
Bustos (2011)	?	+	+	+	+	+
Byrnes et al. (2010)	+	+	+	?	?	−
Bywater et al. (2019)	−	+	?	+	+	+
Chiapa et al. (2015)	?	?	?	?	+	+
Costello et al. (2019)	+	−	+	?	−	−
Eames et al. (2008)	−	?	+	+	+	+
Eames et al. (2009)	−	?	+	?	+	−
Feely et al. (2018)	+	+	+	+	+	−
Forgatch and DeGarmo (2011)	−	+	−	?	+	+
Forgatch et al. (2005)	−	−	+	+	+	+
Giannotta et al. (2019)	+	+	−	+	+	+
Gross et al. (2015)	+	+	+	?	+	−
Hill and Owens (2013)	+	+	+	?	+	+
Hogue et al. (1998)	+	+	+	+	+	+
Hogue et al. (2005)	+	+	+	+	+	−
Hogue et al. (2008)	+	+	+	+	+	−
Hukkelberg and Ogden (2013)	−	+	?	?	?	−
Kogan et al. (2016)	+	?	+	?	?	−
Leer and Lopez-Boo (2019)	?	+	+	?	+	−
Rendu (2004)	?	+	+	?	+	+
Roggman et al. (2001)	?	+	+	?	+	+
Roggman et al. (2016)	+	+	?	+	+	+
Scott et al. (2008)	+	+	+	−	?	−
Shenderovich et al. (2019)	?	+	?	?	+	−
Sigmarsdottir and Guomundsdottir (2013)	?	+	?	+	+	−
Sigmarsdottir et al. (2019)	?	+	?	+	+	−
Singer, 2001)	+	+	+	+	+	+
Smith et al. (2013)	+	+	+	+	+	+
Smith et al. (2015)	+	+	+	?	+	+
Smith et al. (2016)	+	+	+	+	+	+
Smith et al. (2019)	+	+	+	+	+	+
Snider (2019)	+	+	+	?	+	+
Solomon et al. (2014)	+	+	+	?	?	−
St. George et al. (2016)	+	+	+	?	+	−
Sterrett-Hong et al. (2017)	+	−	+	?	?	−
Strauss et al. (2012)	+	?	?	?	?	−
Timmer et al. (2019)	+	?	+	?	?	−
Travis (2012)	−	+	−	?	+	+
Webster-Stratton et al. (2014)	+	+	+	?	+	+

‘+’ refers to ‘met criteria,’ ‘−’ refers to ‘did not meet criteria,’ and ‘?’ refers to ‘insufficient information’

#### Session Sampling

Twenty-four (58.5%) studies had high-quality observation sampling, nine (22.0%) had low-quality observation sampling, and eight (19.5%) did not provide sufficient information. For example, studies were rated as low-quality for allowing facilitators or assessors to select sessions or videos for observation because this permits bias to influence assessments and may inaccurately reflect typical delivery. High-quality studies either observed or rated all sessions or randomly selected sessions for observation, such as in a study by Giannotta et al. (2019) wherein the researchers assessed a random 25.0% of sessions.

#### Assessors

Thirty-two (78.0%) studies were high quality as they used multiple independent assessors, six (14.6%) did not provide sufficient information, and three (7.3%) were low quality as they did not use both multiple and independent assessors.

#### Reactivity

Only a handful of studies explicitly acknowledged facilitator reactivity. However, 28 (68.3%) studies were high quality because all sessions were filmed. Routine use of video reduces reactivity as those being observed habituate to being recorded, thus, minimizing the impact on their behavior (Kazdin, 1982). Of the remaining studies, three (7.3%) were low quality based on information suggesting that reactivity was not well taken into account and 10 (24.4%) did not provide sufficient information.

#### Measure Practicality

Each study’s measure was evaluated using the Measure Practicality Checklist (Table 5). Of the 17 (41.5%) studies providing sufficient information on feasibility and sustainability of training, 16 (94.2%) were deemed high quality due to factors such as having a reasonable number of training hours. Thirty-one (75.6%) studies were rated as high quality on measure utility based on having a measure which was practical to use, nine (22.0%) did not provide sufficient information, and one (2.4%) was low quality due to factors such as the complexity of conducting the assessment. Over half of the studies (51.2%) were rated high quality on availability as they included the measure in the article, or it was easily found online.

### Reliability Results

Twenty (48.8%) studies reported on internal consistency (Table 6). Thirteen studies reported Cronbach Alphas (0.64 to 0.98), eight reported Spearman correlations (− 0.61 to 0.90), and one reported ICCs (0.50 to 0.90). Two (4.9%) studies reported on intra-rater reliability by providing percentage agreements (60.0 to 87.0%) and ICCs (− 0.143 to 0.935). Both studies used one assessor and did not report the interval between assessments. All studies reported on inter-rater reliability. Twenty-one reported ICCs (− 0.03 to 0.96), 17 reported percentage agreements (50.0% to 100.0%), 11 reported Cohen’s Kappas (− 0.01 to 0.97), three reported Pearson’s correlations (− 0.04 to 0.88), one reported Gwet’s ACs (0.85 to 0.91), and one reported a Cronbach Alpha (0.87). Thirteen reported on inter-rater reliability in multiple ways (e.g., percentage agreements and Kappas). No studies reported on test–retest reliability.

**Table 6:** A Summary of the Reliability and Validity Results

Study	Summary of reliability evidence				Summary of validity evidence		
	Internal consistency	Inter-rater reliability	Intra-rater reliability	Test–retest reliability	Content validity	Construct validity	Convergent validity
Askeland et al. (2019)	NR	+	NR	NR	NR	NR	NR
Berkel et al. (2018)	+	−	NR	NR	NR	−	NR
Bustos (2011)	+	?	NR	NR	NR	NR	−
Byrnes et al. (2010)	NR	−	NR	NR	NR	NR	NR
Bywater et al. 2019)	−	−	−	NR	+	NR	NR
Chiapa et al. (2015)	+	+	NR	NR	NR	NR	NR
Costello et al. (2019)	NR	?	NR	NR	NR	NR	NR
Eames et al. (2008)	+	+	+	NR	NR	NR	NR
Eames et al. (2009)	NR	?	NR	NR	NR	NR	NR
Feely et al. (2018)	NR	+	NR	NR	?	NR	NR
Forgatch and DeGarmo (2011)	+	+	NR	NR	NR	+	NR
Forgatch et al. (2005)	+	+	NR	NR	NR	+	NR
Giannotta et al. (2019)	NR	+	NR	NR	NR	+	NR
Gross et al. (2015)	+	+	NR	NR	NR	NR	NR
Hill and Owens (2013)	NR	+	NR	NR	NR	NR	NR
Hogue et al. (1998)	−	+	NR	NR	?	−	NR
Hogue et al. (2005)	−	−	NR	NR	NR	−	NR
Hogue et al. (2008a)	−	−	NR	NR	NR	NR	NR
Hukkelberg and Ogden (2013)	+	−	NR	NR	NR	NR	NR
Kogan et al. (2016)	NR	+	NR	NR	NR	NR	NR
Leer and Lopez-Boo (2019)	NR	?	NR	NR	NR	NR	NR
Rendu (2004)	NR	?	NR	NR	NR	−	NR
Roggman et al. (2001)	NR	+	NR	NR	NR	NR	NR
Roggman et al. (2016)	−	+	NR	NR	NR	NR	NR
Scott et al. (2008)	−	−	NR	NR	NR	−	NR
Shenderovich et al. (2019)	+	+	NR	NR	NR	NR	NR
Sigmarsdottir and Guomundsdottir (2013)	+	+	NR	NR	NR	NR	NR
Sigmarsdottir et al. (2019)	NR	+	NR	NR	NR	NR	NR
Singer (2001)	NR	+	NR	NR	NR	NR	NR
Smith et al. (2013)	−	+	NR	NR	NR	+	NR
Smith et al. (2015)	NR	+	NR	NR	NR	NR	NR
Smith et al. (2016)	+	−	NR	NR	NR	NR	NR
Smith et al. (2019)	+	−	NR	NR	NR	NR	NR
Snider, 2019)	NR	+	NR	NR	NR	NR	NR
Solomon et al. (2014)	NR	+	NR	NR	NR	NR	NR
St. George et al. (2016)	NR	?	NR	NR	NR	NR	NR
Sterrett-Hong et al. (2017)	NR	+	NR	NR	NR	NR	NR
Strauss et al. (2012)	NR	−	NR	NR	NR	NR	NR
Timmer et al. (2019)	NR	+	NR	NR	NR	NR	NR
Travis (2012)	NR	+	NR	NR	NR	NR	NR
Webster-Stratton et al. (2014)	NR	?	NR	NR	NR	NR	NR

‘+’ refers to ‘met criteria,’ ‘−’ refers to ‘did not meet criteria,’ ‘?’ refers to ‘insufficient information,’ and *NR* not reported

Based on the Measure Risk of Bias and Quality Checklist results (Table 5), 21 (51.2%) of the 41 studies reporting inter-rater reliability were high quality (ICC > 0.70; Kappa > 0.70; r > 0.80). Of the two studies reporting intra-rater reliability, one was high quality (ICC > 0.70). Of the 20 (48.8%) studies reporting internal consistency, 11 (63.0%) were high quality (Cronbach Alpha > 0.70).

### Validity Results

Only three (7.3%) studies reported on content validity (Table 6). Of these, two indicated using the knowledge and advice of experts to develop the measure (Feely et al., 2018; Hogue et al., 1998), two reported asking experts to assess a draft measure (Feely et al., 2018; Hogue et al., 1998), one described seeking feedback from program trainers about the developed measure (Bywater et al., 2019), and one asked a program developer to review their draft measure (Hogue et al., 1998). They all provided very little detail about the process, feedback, or use of feedback.

Nine (22.0%) studies reported on construct validity (Tables 6 and 7). Four performed confirmatory factor analyses, three performed principal components factor analyses, one performed principal axis factor analysis, and one performed exploratory factor analysis. One (2.4%) study assessed convergent validity, reporting interscale correlations among the measure of interest and similar measures with Pearson’s correlations that were statistically significant at the *p* < 0.01 level ranging from 0.35 to 0.55. No studies reported on divergent validity or criterion validity.

**Table 7:** The construct validity findings extracted

Study citation	Type of factor analysis	Model fit	Factor loading	Number constructs or factors	Percentage of variance	Eigenvalues	Other
Berkel et al. (2018)	Confirmatory factor analysis	*χ*^2^ (247) = 753.36, *p* ≤ 0.001, RMSEA = 0.07 (0.06–0.07), SRMR = 0.06, CFI = 0.90	All items loaded significantly on their subscales, *p* ≤ 0.001	Not reported	Not reported	Not reported	Not applicable
Forgatch and DeGarmo (2011)	Principal components factor analysis	Not reported	Not reported	Obtained a single-factor solution	Not reported	4.67, 4.66, and 4.58 at each of the time points	Not applicable
Forgatch et al. (2005)	Confirmatory factor analysis	Not reported	Not reported	Obtained a single-factor solution (encouragement and discipline)	Encouragement—66%, discipline—85%, one-factor solution—53%	Encouragement—3.3, discipline 4.3, one-factor solution—5.3	Not applicable
Giannotta et al. (2019)	Confirmatory factor analysis	*χ*^22^(4) = 3.62, *p* > 0.05, CFI = 1.0, RMSEA = 0.00, SRMR = 0.01	Not reported	Obtained a single-factor solution (adherence and competence)	Not reported	Not reported	Not applicable
Hogue et al. (1998)	Confirmatory factor analysis	Not reported	Not reported	Obtained a four-factor solution (modality, affect/system focused, behavior/skillsfocused, cognition focused)	Modality—15%, affect/system focused—12%, behavior/skills focused—11%, cognition focused—8%	Modality—5.16, affect/system focused—2.88, behavior/skills focused—2.25, cognition focused—1.82	Kaiser–meyer–olkin measure of sampling adequacy was 0.64
Hogue et al. (2005)	Exploratory factor analysis	Not reported	Factor loadings ranged from 0.39 to 0.84	A three-factor solution was strongest and accounted for 39% of total variance	Behavior/cognition scale—17%, affect/systems scale—14%, monitoring/knowledge scale—8%	Behavior/cognition—3.34, affect/systems scale—2.81, monitoring/knowledge scale—1.67	Kaiser–meyer–olkin measure of sampling adequacy was 0.68
Rendu (2004)	Principal components factor analysis	Not reported	Not reported	Obtained a two-factor solution from the eight variables	Not reported	Not reported	Table 5 Varimax rotated factor loadings
Scott et al. (2008)	Principal components factor analysis	Not reported	Table 2 (10 different factor loadings reported)	Obtained a two-factor solution (therapist skill and organization)	Therapist skill—50%, Therapist organization—21%	Not reported	Kaiser–meyer–olkin measure of sampling adequacy was acceptable at 0.57; Bartlett's test of sphericity was highly significant (*p* < 0.0001)
Smith et al. (2013)	Principal axis factor analysis	Not reported	Factor loadings ranged from 0.54 to 0.79	Obtained a one-factor solution	Not reported	Not reported	Not applicable

Each study was evaluated using the Measure Risk of Bias Checklist (Table 5). The one study that reported on convergent validity did not meet the quality criteria as correlations between the scales were not all above 0.50. Of the three reporting on content validity, one was high quality and two did not provide sufficient information. The studies reporting on content validity provided insufficient details about the process, feedback, and use of the feedback to make an evaluation of the study’s content validity. Of the nine studies reporting on construct validity, four were high-quality and five were low-quality.

## Discussion

### Summary of Findings

#### Measurement Characteristics

Our review provides the first synthesis and critical analysis of the range of tools used to measure facilitator competent adherence in the parenting field. The majority of studies included assessments of adherence and more than half reported measuring competence. Video-based observation (alone or in combination) was the most common assessment method, demonstrating that most assessors used a rigorous mode of data collection. Among non-observational methods, memory-based assessments were most common with most being facilitator self-reports. However, in some cases, parents or supervisors were asked to recount facilitator delivery. Assessments relying on memory are less rigorous since their reliability is weaker for reasons including that memory can be faulty and, in the case of self-reports, facilitators may record socially desirable responses (Stone et al., 2000). Among studies reporting on assessors types, facilitators and external assessors (e.g., researchers, trainers) were most frequently reported.

All studies measured adherence, mostly with dichotomous items. Competence was largely recorded using Likert items. It was predicted that more measures would report dichotomous items due to their simplicity and reliability. However, the finding that most measures used Likert items suggests an attempt to capture the complexity of delivery.

Assessor training was rarely reported, yet it is a key element of a tool’s success and provides information about the resources required (Girard & Cohn, 2016). Of the 52 studies reporting on training, a third described the amount of training provided. Further, all of the training reported was for third-party assessors, such as researchers and program staff, and none was for facilitators self-assessing their delivery. Further, training was most commonly provided to assessors of observational measures. This is a strength in the literature as training is the main way to ensure reliability (Multon & Colemon, 2018). However, the finding also signals a further limitation in the literature on non-observational measures (e.g., self-assessment), as they are already less reliable, and a lack of training may exacerbate their unreliability. At last, only a minority of the training was provided for assessors of adherence-only measures, perhaps because they are generally considered simpler to administer. However, simplicity should not be assumed, especially when reliability (e.g., intra- and inter-rater reliability) has not yet been established.

#### Measure Types

The dominant types of measures identified in Part One of the review can be illustrated using four conceptual groupings. The first group measures adherence by asking facilitators to self-report using dichotomous items. An example of such a non-observational tool is used by Lester (2015) in the Positive Parenting Skills Training Program wherein facilitators complete session-specific forms with 11–12 ‘yes’ or ‘no’ questions. A strength of this type of measure is that it is quick and simple yet, reliability may be questionable due to factors such as social desirability.

The second type involves researchers or supervisors completing video or live assessments of competent adherence. A study by Bywater et al. (2019) on the Incredible Years, for instance, reports on the Parent Program Implementation Checklist used to capture competent adherence. This checklist is composed of 18 items rated from ‘not at all’ (1 point) to ‘excellent’ (5 points). Although this approach provides rich and objective information about facilitator delivery, it is time consuming and resource intensive.

The third type is an observational and/or non-observational measure that uses multiple assessors to capture one or more aspects of competent adherence. An example is the Alternatives for Families: A CBT Program Treatment Adherence Form described by Herschell et al., (2019). In this study, three different assessor types were used—caregivers, facilitators, and experts. Caregivers and facilitators recorded their recollection of delivery whereas experts used audio recordings. All assessors were asked to complete nine dichotomous items (‘occurrence’ or ‘non-occurrence’) to indicate facilitator adherence. The use of multiple assessors not only may enhance the reliability of the assessments, but it also requires considerably more time and effort to analyse and use the results, especially if there is disagreement among assessors.

The fourth type is a non-observational measure that asks participants to assess one or more aspects of facilitator competent adherence. For instance, Chapman et al. (2011) asked families in MultiSystemic Therapy to rate adherence using the Revised Treatment Adherence Measure. This measure captures nine aspects of delivery using 28 items rated on a five-point scale from ‘not at all’ to ‘very much.’ Using participant assessors is valuable in that it captures an important perspective. However, reliability may be limited due to factors such as relying on memory and not receiving training on how to conduct assessments.

#### Missing or Under-Reported Measurement Characteristics

It is also important to note the measure characteristics absent from the literature. Only one study indicated how long it took to complete an assessment (10 minutes) and none reported the cost of assessment or training. Time and cost information would be valuable for future research teams to ensure they understand the resources necessary. Additionally, some measurement characteristics were underreported. For example, over half of the studies did not report on assessor training, almost three-quarters did not provide the measure(s) used, and nearly all studies did not report on how measures were completed. This lack of detail speaks to a general under-reporting of how measures are implemented in practice.

#### Facilitator Characteristics

The facilitator characteristics of interest were not consistently reported. The limited data indicated that facilitators were mostly small samples of highly educated and experienced Caucasian females of various ages, with a few studies reporting African-American facilitators. More than half the studies reported sample sizes under 30, and few had sample sizes greater than 100. There was also limited research available on the use of paraprofessionals or lay facilitators, such as community workers. These findings may suggest that the intra- and inter-rater reliability results are inflated as facilitators delivering parenting programs at scale, particularly in LMICs, may have less experience and education than those reported in this review. Additionally, the small samples weaken the generalizability of findings.

#### Intervention Characteristics

An analysis of intervention characteristics reveals that most studies were conducted in the USA and other high-income countries, particularly in Northern Europe, and only eight of 156 in LMICs. Thus, there is a paucity of research on competent adherence in LMICs, consistent with there being fewer evaluations of parenting programs in LMICs (Knerr et al., 2013).

### Quantity and Quality of Psychometric Literature

Part Two of the review found limited psychometric evidence on measures of facilitator competent adherence. Of the 41 studies analyzed, the only psychometric property reported consistently was inter-rater reliability. Internal consistency was the next most frequently (48.8%) reported property, with only 22.2% reporting on construct validity, 7.3% on content validity, 4.9% on intra-rater reliability, 2.4% on convergent validity, and none on test–retest reliability or criterion validity. Despite the limited evidence, most measures had moderate reliability and validity, with the measures reported in more than one study being the most robust.

#### Overall Reliability

Based on the Measure Risk of Bias and Quality Checklist, the intra- and inter-rater reliability results appear mixed, but the internal consistency results largely appear positive. However, upon further consideration, a more nuanced perspective is required as the results generated by the COSMIN checklist alone do not tell the full story. The assessments of studies reporting on inter-rater reliability indicated that only 51.2% met the criteria (ICCs and Kappa’s above 0.70 or Pearson’s correlations above 0.80). While this suggests that assessors were inconsistent in their understanding and application of items, a considerable number of results were very close to the quality cut-offs or produced findings both above and below the quality threshold. For instance, Byrnes et al. (2010) reported a mean percentage agreement of 92.0% and a Cohen’s Kappa of 0.67 (only 0.03 from the 0.70 cut-off), suggesting that the measure is not properly assessed as entirely unreliable based on an absolute threshold. Further, a number of studies were not rated as high quality as they only reported unadjusted percentage agreements. Yet, all reported agreements above 70.0%—a level many researchers consider sufficient (Aspland & Gardner, 2003). Further, when using the rigorous COSMIN standards, only reporting percentage agreements is insufficient as this index does not take chance agreement into account (McHugh, 2012). Thus, the findings suggest that more research is necessary to improve inter-reliability, especially as there is heavy reliance on percentage agreements.

Intra-rater reliability was rarely reported (4.9%), and in those studies reporting, the findings were mixed. One study met the quality criteria and one did not. That the percentage agreement was not always above 80.0% in one study and that ICCs were highly variable in another suggests that the assessors were not applying the measure consistently over time. This may indicate that assessor understanding of items changed or that assessors weighed different considerations due to poor item clarity and/or training deficiencies (Multon & Colemon, 2018).

Twenty (48.8%) studies reported on internal consistency, with more than half rated as high quality. Among low-quality studies, those reporting Cronbach Alphas all had results close to the quality threshold. For instance, Bywater et al. (2019) found the internal consistency of the adherence subscale to be 0.66, competence subscale to be 0.78, and overall scale to be 0.82. This indicates sufficient reliability as only the adherence subscale did not meet the 0.70 quality threshold and the overall results were above the threshold. Among low-quality studies reporting correlations, results were more varied. For example, Eames et al. (2008) found a wide range of correlations. The finding that 12 studies were high quality and five were borderline indicates that measures had better consistency than the checklist results indicate.

As previously noted, no studies examined test–retest reliability. Test–retest reliability is a valuable property to examine in future research; filling this gap will provide insight into whether facilitator assessment results are representative of their overall performance.

#### Overall Validity

Only 12 (29.3%) studies reported on validity. From analyzing the Measure Risk of Bias and Quality Checklist results, the validity of the measures appears mediocre. However, the validity of studies deemed low quality is arguably better than the COSMIN checklist ratings indicate. Ideally, assessments of content validity would describe the overall purpose and intended use of the measure, who was involved in creating items, how items were constructed, who was asked to provide feedback on the measure and in what format (e.g., Delphi method), and how feedback was used (Fish & Busby, 1996; Terwee et al., 2018). Content validity requires substantial future attention since only three (7.3%) studies provided information about this property, two of which did not provide sufficient information. The study by Feely et al. (2018) was deemed high quality because detail was provided, the measure was developed based on program components, and experts supported and reviewed the measure. Overall, the lack of reporting indicates that our understanding of the foundation on which measures have been built is unclear.

Of the nine (22.2%) studies reporting construct validity, four were high quality (e.g., factors explaining at least 50.0% of the variance) and five were low quality (Mokkink et al., 2010b). Among the latter, factor loadings were close to the threshold in many of the studies, with some above and below the cut-off and other indicators (e.g., model fit) close to those considered high quality. For example, a study by Berkel et al. (2018) was assessed as low quality with a comparative fit index of 0.90—which still represents quite good model fit. Despite over half of the studies not meeting COSMIN criteria, the measures are mostly capturing their intended constructs providing evidence that assessment results can be trusted. Thus, the results indicate that measure assessments can be used to enhance implementation practices and processes.

Only one (2.4%) study reported on convergent validity and it did not meet the quality criteria (correlations above 0.50), but it did have some correlations above and below the threshold. Researchers should not seek to rectify this paucity of research until the literature is substantial enough to permit comparisons between measures.

None of the studies reported on criterion validity. This finding is in keeping with our observation that there is no gold standard in the literature. Once there is more evidence on measure psychometric properties, a gold standard measure may emerge.

### Overall Study Quality and Measure Practicality

The risk of bias and quality assessment results suggest that studies are of moderate quality. Further, an evaluation of measure practicality suggests that, among the studies providing sufficient information, the measures are moderately practical to use. It is fundamental that interventions and their components are practical, particularly at scale (Fixsen et al., 2019).

### Strengths and Limitations

This systematic review has a number of strengths. It is the first to synthesize and critically appraise both measures of competent adherence and the psychometric properties of observational measures in the parenting program literature. The high inter-rater reliability achieved between coders suggests minimal human error in screening and data extraction. Moreover, a checklist was created wherein a number of considerations were added to the COSMIN guidelines to take the nature and challenges of observational measurement into account. Further, questions were created to assess the usability of measures in practice. On the other hand, the review was limited by the paucity of evidence on the psychometric properties of measures and this lack of data hinders strongly supported findings from being made. Additionally, measures of facilitator competent adherence are often program specific. As a result, the review’s ability to recommend the selection of certain measures for broader use is limited. However, the review provides critical information to help guide the adaptation and development of future measures and to conclude that assessments of facilitator competent adherence are being made based on measures with limited psychometric evidence.

### Recommendations for Future Research and Practice

Findings from this review suggest a number of recommendations for future research and practice. Reporting guidelines for studies reporting on fidelity measures should be created so that measure characteristics (e.g., method of completion, assessors) and psychometric properties, as well as essential facilitator and intervention characteristics, are consistently documented. Reporting guidelines would advance the knowledge generated by reducing reporting bias and providing a framework from which to evaluate measure validity and reliability. The findings also suggest that there are a variety of facilitator characteristics that need exploring in future studies to determine if outcomes are associated with facilitator characteristics and levels of competent adherence. Reporting guidelines would allow researchers to learn from each other in order to create, modify, and use high-quality measures of competent adherence in research and practice.

A subsequent systematic review exploring the predictive validity of facilitator competent adherence measures is recommended. Such a review would reveal whether increased facilitator competent adherence is associated with better parent and child program outcomes. The results of this further meta-analysis may contribute to an eventual clarification of the mixed evidence regarding the association between facilitator delivery and participant outcomes.

More rigorous research on measures of facilitator competent adherence should be conducted, particularly as it relates to reliability and validity. Findings as to whether measures of facilitator competent adherence are reliable and valid will help establish if assessment results can be relied on, enhance facilitator delivery, and determine whether competent adherence is correlated with outcomes for program beneficiaries (Fixsen et al., 2005). In addition, more studies of competent adherence are warranted in LMICs. The need for low-cost parenting programs is greatest in LMICs, thus, heightening the necessity for solid evidence on improving program delivery and outcomes (Mercy et al., 2008).

## Conclusion

This two-part review summarizes and evaluates measures of facilitator competent adherence in the parenting program literature. The findings further knowledge about and identify gaps regarding the competent adherence with which facilitators deliver parenting programs. This is an essential area of intervention science research to advance as facilitators play a critical role in program implementation. Ensuring assessments of facilitator delivery are captured accurately, consistently, and to a high level of quality will support future determinations as to whether decisions made based on assessments using these measures are evidence-based.

## Supplementary Information

Below is the link to the electronic supplementary material.

Supplementary file1 (DOCX 61 KB)

Supplementary file2 (DOCX 27 KB)

Supplementary file3 (DOCX 31 KB)

## Data Availability

Supplementary Materials 1–3.
