# Supplementary material for: Measures of Facilitator Competent Adherence Used in Parenting Programs and Their Psychometric Properties: A Systematic Review
Source: Clin Child Fam Psychol Rev. 2021 May 21;24(4):834–53. doi: 10.1007/s10567-021-00350-8 (PMC8541983; doi:10.1007/s10567-021-00350-8)
Supplement: Supplementary file 1 — Supplementary file1 (DOCX 61 KB) [file 10567_2021_350_MOESM1_ESM.docx]

**Measures of Facilitator Competent Adherence used in Parenting Programs and their Psychometric Properties: A Systematic Review**

Mackenzie Martin, Bridget Steele, Jamie M. Lachman, and Frances Gardner

Department of Social Policy and Intervention

University of Oxford, United Kingdom

Correspondence regarding this article should be addressed to Mackenzie Martin, Department of Social Policy and Intervention, University of Oxford

Email: Mackenzie.Martin@spi.ox.ac.uk

# Studies Included in the Review

# Part 1

1. Aarons, G. A., Miller, E. A., Green, A. E., Perrott, J. A., & Bradway, R. (2012). Adaptation happens: A qualitative case study of implementation of the incredible years evidence‐based parent training programme in a residential substance abuse treatment programme. *Journal of Children's Services*.
2. Aarons, G. A., Sommerfeld, D. H., Hecht, D. B., Silovsky, J. F., & Chaffin, M. J. (2009). The impact of evidence-based practice implementation and fidelity monitoring on staff turnover: Evidence for a protective effect. *Journal of Consulting and Clinical Psychology, 77*(2), 270.
3. Agazzi, H., Adams, C., Ferron, E., Ferron, J., Shaffer-Hudkins, E., & Salloum, A. (2019). Trauma-informed behavioral parenting for early intervention. *Journal of Child and Family Studies, 28*(8), 2172-2186.
4. Álvarez, M., Rodrigo, M. J., & Byrne, S. (2018). What implementation components predict positive outcomes in a parenting program? *Research on Social Work Practice, 28*(2), 173-187.
5. Amorós-Martí, P., Byrne, S., Mateos-Inchaurrondo, A., Vaquero-Tió, E., & Mundet-Bolós, A. (2016). “Learning together, growing with family”: The implementation and evaluation of a family support programme. *Psychosocial Intervention, 25*(2), 87-93.
6. Asgary-Eden, V., & Lee, C. M. (2011). So now we've picked an evidence-based program, what's next? Perspectives of service providers and administrators. *Professional Psychology: Research and Practice, 42*(2), 169.
7. Askeland, E., Forgatch, M. S., Apeland, A., Reer, M., & Grønlie, A. A. (2019). Scaling up an empirically supported intervention with long-term outcomes: The nationwide implementation of generation pmto in Norway. *Prevention Science, 20*(8), 1189-1199.
8. Bearss, K., Burrell, T. L., Challa, S. A., Postorino, V., Gillespie, S. E., Crooks, C., & Scahill, L. (2018). Feasibility of parent training via telehealth for children with autism spectrum disorder and disruptive behavior: A demonstration pilot. *Journal of Autism and Developmental Disorders, 48*(4), 1020-1030.
9. Bearss, K., Lecavalier, L., Minshawi, N., Johnson, C., Smith, T., Handen, B., Sukhodolsky, D., Aman, M., Swiezy, N., & Butter, E. (2013). Toward an exportable parent training program for disruptive behaviors in autism spectrum disorders. *Neuropsychiatry, 3*(2), 169.
10. Beasley, L. O., Silovsky, J. F., Owora, A., Burris, L., Hecht, D., DeMoraes-Huffine, P., Cruz, I., & Tolma, E. (2014). Mixed-methods feasibility study on the cultural adaptation of a child abuse prevention model. *Child Abuse & Neglect, 38*(9), 1496-1507.
11. Berkel, C., Mauricio, A. M., Sandler, I. N., Wolchik, S. A., Gallo, C. G., & Brown, C. H. (2018). The cascading effects of multiple dimensions of implementation on program outcomes: A test of a theoretical model. *Prevention Science, 19*(6), 782-794.
12. Bloomquist, M. L., August, G. J., Lee, S. S., Lee, C.-Y. S., Realmuto, G. M., & Klimes-Dougan, B. (2013). Going-to-scale with the early risers conduct problems prevention program: Use of a comprehensive implementation support (cis) system to optimize fidelity, participation and child outcomes. *Evaluation and Program Planning, 38*, 19-27.
13. Bloomquist, M. L., Horowitz, J. L., August, G. J., Lee, C.-Y. S., Realmuto, G. M., & Klimes-Dougan, B. (2009). Understanding parent participation in a going-to-scale implementation trial of the early risers conduct problems prevention program. *Journal of Child and Family Studies, 18*(6), 710-718.
14. Breitenstein, S. M., Fogg, L., Garvey, C., Hill, C., Resnick, B., & Gross, D. (2010). Measuring implementation fidelity in a community-based parenting intervention. *Nursing Research, 59*(3), 158.
15. Bustos, C. E. (2011). Parent experiences of a family-centered intervention: Examining ethnocultural group differences. [Doctoral dissertation, University of Oregon]. https://scholarsbank.uoregon.edu/xmlui/handle/1794/11228
16. Byrnes, H. F., Miller, B. A., Aalborg, A. E., Plasencia, A. V., & Keagy, C. D. (2010). Implementation fidelity in adolescent family-based prevention programs: Relationship to family engagement. *Health Education Research, 25*(4), 531-541.
17. Bywater, T., Gridley, N., Berry, V., Blower, S., & Tobin, K. (2019). The parent programme implementation checklist (ppic): The development and testing of an objective measure of skills and fidelity for the delivery of parent programmes. *Child Care in Practice, 25*(3), 281-309.
18. Cantu, A. M., Hill, L. G., & Becker, L. G. (2010). Implementation quality of a family-focused preventive intervention in a community-based dissemination. *Journal of Children's Services, 5*(4), 18.
19. Chapman, J. E., & Schoenwald, S. K. (2011). Ethnic similarity, therapist adherence, and long-term multisystemic therapy outcomes. *Journal of Emotional and Behavioral Disorders, 19*(1), 3-16.
20. Chase, R. M., Carmody, K. A., Lent, M., Murphy, R., Amaya-Jackson, L., Wray, E., Ake III, G. S., Sullivan, K., White, D., & Gurwitch, R. (2019). Disseminating parent-child interaction therapy through the learning collaborative model on the adoption and implementation of an evidence-based treatment. *Children and Youth Services Review, 101*, 131-141.
21. Chiapa, A., Smith, J. D., Kim, H., Dishion, T. J., Shaw, D. S., & Wilson, M. N. (2015). The trajectory of fidelity in a multiyear trial of the family check-up predicts change in child problem behavior. *Journal of Consulting and Clinical Psychology, 83*(5), 1006.
22. Costello, A. H., Roben, C. K., Schein, S. S., Blake, F., & Dozier, M. (2019). Monitoring provider fidelity of a parenting intervention using observational methods. *Professional Psychology: Research and Practice, 50*(4), 264.
23. Coutts, M. J. (2015). Conjoint behavioral consultation via distance delivery (cbc-d): An evaluation of efficacy and acceptability. *Public Access Theses and Dissertations from the College of Education and Human Sciences.*
24. Czymoniewicz-Klippel, M. T., Chesnut, R. P., DiNallo, J., & Perkins, D. F. (2017). Understanding the implementation of the grow! Parenting program: Findings from a mixed methods pilot study. *Children and Youth Services Review, 82*, 99-107.
25. David, P., & Schiff, M. (2018). Initial clinician reports of the bottom-up dissemination of an evidence-based intervention for early childhood trauma. *Child & Youth Care Forum.*
26. Eames, C., Daley, D., Hutchings, J., Hughes, J., Jones, K., Martin, P., & Bywater, T. (2008). The leader observation tool: A process skills treatment fidelity measure for the incredible years parenting programme. *Child: Care, Health and Development, 34*(3), 391-400.
27. Eames, C., Daley, D., Hutchings, J., Whitaker, C., Jones, K., Hughes, J., & Bywater, T. (2009). Treatment fidelity as a predictor of behaviour change in parents attending group‐based parent training. *Child: Care, Health and Development, 35*(5), 603-612.
28. Eames, C., Daley, D., Hutchings, J., Whitaker, C. J., Bywater, T., Jones, K., & Hughes, J. C. (2010). The impact of group leaders’ behaviour on parents acquisition of key parenting skills during parent training. *Behaviour Research and Therapy, 48*(12), 1221-1226.
29. Edginton, E., Walwyn, R., Burton, K., Cicero, R., Graham, L., Reed, S., Tubeuf, S., Twiddy, M., Wright-Hughes, A., & Ellis, L. (2017). Tiga-cub–manualised psychoanalytic child psychotherapy versus treatment as usual for children aged 5–11 years with treatment-resistant conduct disorders and their primary carers: Study protocol for a randomised controlled feasibility trial. *Trials, 18*(1), 431.
30. Edwards, G. (2018). Behavioural parent training in children with autism: Feasibility and effectiveness of a group intervention. *ProQuest Dissertations & Theses Global*.
31. Ellis, M. L., Weiss, B., Han, S., & Gallop, R. (2010). The influence of parental factors on therapist adherence in multi-systemic therapy. *Journal of Abnormal Child Psychology, 38*(6), 857-868.
32. Feely, M., Seay, K. D., Lanier, P., Auslander, W., & Kohl, P. L. (2018). Measuring fidelity in research studies: A field guide to developing a comprehensive fidelity measurement system. *Child and Adolescent Social Work Journal, 35*(2), 139-152.
33. Fettig, A., & Barton, E. E. (2014). Parent implementation of function-based intervention to reduce children’s challenging behavior: A literature review. *Topics in Early Childhood Special Education, 34*(1), 49-61.
34. Forgatch, M. S., & DeGarmo, D. S. (2011). Sustaining fidelity following the nationwide pmto™ implementation in norway. *Prevention Science, 12*(3), 235-246.
35. Forgatch, M. S., Patterson, G. R., & DeGarmo, D. S. (2005). Evaluating fidelity: Predictive validity for a measure of competent adherence to the Oregon model of parent management training. *Behavior Therapy, 36*(1), 3-13.
36. Furlong, M. (2013). Implementing the incredible years parenting programme in disadvantaged settings in Ireland: A process evaluation. [Doctoral dissertation, National University of Ireland Maynooth]. <https://www.incredibleyears.com/article/implementing-the-incredible-years-parenting-programme-in-disadvantaged-settings-in-ireland-a-process-evaluation/>.
37. Furlong, M., & McGilloway, S. (2015). Barriers and facilitators to implementing evidence-based parenting programs in disadvantaged settings: A qualitative study. *Journal of Child and Family Studies, 24*(6), 1809-1818.
38. Galanter, R., Self-Brown, S., Valente, J. R., Dorsey, S., Whitaker, D. J., Bertuglia-Haley, M., & Prieto, M. (2012). Effectiveness of parent–child interaction therapy delivered to at-risk families in the home setting. *Child & Family Behavior Therapy, 34*(3), 177-196.
39. Garbacz, L. L., Brown, D. M., Spee, G. A., Polo, A. J., & Budd, K. S. (2014). Establishing treatment fidelity in evidence-based parent training programs for externalizing disorders in children and adolescents. *Clinical Child and Family Psychology Review, 17*(3), 230-247.
40. Giannotta, F., Özdemir, M., & Stattin, H. (2019). The implementation integrity of parenting programs: Which aspects are most important? *Child and Youth Care Forum*, *48*, 917-933.
41. Gillespie, M. L., Huey Jr, S. J., & Cunningham, P. B. (2017). Predictive validity of an observer-rated adherence protocol for multisystemic therapy with juvenile drug offenders. *Journal of Substance Abuse Treatment, 76*, 1-10.
42. Gilman, L. (2009). *Supervisory interventions and treatment adherence: An observational study of supervisor interventions and their impact on therapist model* adherence. *.* [Doctoral dissertation, Indiana University]. <https://search.proquest.com/openview/ee5dacb0c58f831f4b1b83998ae5e6fc/1?pq-origsite=gscholar&cbl=18750&diss=y>
43. Graham, C., Carr, A., Rooney, B., Sexton, T., & Wilson Satterfield, L. R. (2014). Evaluation of functional family therapy in an irish context. *Journal of Family Therapy, 36*(1), 20-38.
44. Gross, T., Alex Mason, W., Parra, G., Oats, R., Ringle, J. L., & Haggerty, K. (2015). Adherence and dosage contributions to parenting program quality. *Journal of the Society for Social Work and Research, 6*(4), 467-489.
45. Hanes, C. W. (2012). Therapist model adherence from the family's perspective: Examining the relationship between adherence and outcome in functional family therapy. [Doctoral dissertation, Indiana University]. <https://search.proquest.com/openview/1809d5a142bdd0458a6a85b8774b0c17/1?pq-origsite=gscholar&cbl=18750&diss=y>
46. Hartnett, D., Carr, A., & Sexton, T. (2016). The effectiveness of functional family therapy in reducing adolescent mental health risk and family adjustment difficulties in an Irish context. *Family Process, 55*(2), 287-304.
47. Henggeler, S. W., Melton, G. B., Brondino, M. J., Scherer, D. G., & Hanley, J. H. (1997). Multisystemic therapy with violent and chronic juvenile offenders and their families: The role of treatment fidelity in successful dissemination. *Journal of Consulting and Clinical Psychology, 65*(5), 821.
48. Henggeler, S. W., Pickrel, S. G., & Brondino, M. J. (1999). Multisystemic treatment of substance-abusing and-dependent delinquents: Outcomes, treatment fidelity, and transportability. *Mental Health Services Research, 1*(3), 171-184.
49. Henggeler, S. W., Schoenwald, S. K., Liao, J. G., Letourneau, E. J., & Edwards, D. L. (2002). Transporting efficacious treatments to field settings: The link between supervisory practices and therapist fidelity in mst programs. *Journal of Clinical Child and Adolescent Psychology, 31*(2), 155-167.
50. Herschell, A. D., Kolko, D. J., Scudder, A. T., Taber-Thomas, S., Schaffner, K. F., Hiegel, S. A., Iyengar, S., Chaffin, M., & Mrozowski, S. (2015). Protocol for a statewide randomized controlled trial to compare three training models for implementing an evidence-based treatment. *Implementation Science, 10*(1), 133.
51. Herschell, A. D., Quetsch, L. B., & Kolko, D. J. (2019). Measuring adherence to key teaching techniques in an evidence-based treatment: A comparison of caregiver, therapist, and behavior observation ratings. *Journal of Emotional and Behavioral Disorders*.
52. Heywood, C., & Fergusson, D. (2016). A pilot study of functional family therapy in New Zealand. *New Zealand Journal of Psychology (Online), 45*(3), 12.
53. Hickey, G., McGilloway, S., Furlong, M., Leckey, Y., Bywater, T., & Donnelly, M. (2016). Understanding the implementation and effectiveness of a group-based early parenting intervention: A process evaluation protocol. *BMC Health Services Research, 16*(1), 490.
54. Hill, L. G., & Owens, R. W. (2013). Component analysis of adherence in a family intervention. *Health Education*, *113*(4), 264-280.
55. Hogue, A., & Dauber, S. (2013). Assessing fidelity to evidence-based practices in usual care: The example of family therapy for adolescent behavior problems. *Evaluation and Program Planning, 37*, 21-30.
56. Hogue, A., Dauber, S., & Henderson, C. E. (2014). Therapist self-report of evidence-based practices in usual care for adolescent behavior problems: Factor and construct validity. *Administration and Policy in Mental Health and Mental Health Services Research, 41*(1), 126-139.
57. Hogue, A., Dauber, S., & Henderson, C. E. (2017). Benchmarking family therapy for adolescent behavior problems in usual care: Fidelity, outcomes, and therapist performance differences. *Administration and Policy in Mental Health and Mental Health Services Research, 44*(5), 626-641.
58. Hogue, A., Dauber, S., Henderson, C. E., & Liddle, H. A. (2014). Reliability of therapist self-report on treatment targets and focus in family-based intervention. *Administration and Policy in Mental Health and Mental Health Services Research, 41*(5), 697-705.
59. Hogue, A., Dauber, S., Lichvar, E., Bobek, M., & Henderson, C. E. (2015). Validity of therapist self-report ratings of fidelity to evidence-based practices for adolescent behavior problems: Correspondence between therapists and observers. *Administration and Policy in Mental Health and Mental Health Services Research, 42*(2), 229-243.
60. Hogue, A., Henderson, C., Dauber, S., Barajas, P., Fried, A., & Liddle, H. (2008). Treatment adherence, competence, and outcome in individual and family therapy for adolescent behavior problems. *Journal of Consulting and Clinical Psychology, 76*(4), 544.
61. Hogue, A., Liddle, H. A., & Rowe, C. (1996). Treatment adherence process research in family therapy: A rationale and some practical guidelines. *Psychotherapy: Theory, Research, Practice, Training, 33*(2), 332.
62. Hogue, A., Liddle, H. A., Rowe, C., Turner, R. M., Dakof, G. A., & LaPann, K. (1998). Treatment adherence and differentiation in individual versus family therapy for adolescent substance abuse. *Journal of Counseling Psychology, 45*(1), 104.
63. Hogue, A., Liddle, H. A., Singer, A., & Leckrone, J. (2005). Intervention fidelity in family‐based prevention counseling for adolescent problem behaviors. *Journal of Community Psychology, 33*(2), 191-211.
64. Hornell, L. (2007). Implementation evaluation of best start for babies. [Doctoral dissertation, Colorado State University Libraries]. <https://mountainscholar.org/bitstream/handle/10217/82555/Hornell_colostate_0053N_12251.pdf?sequence=>
65. Howe, T. R., Knox, M., Altafim, E. R. P., Linhares, M. B. M., Nishizawa, N., Fu, T. J., Camargo, A. P. L., Ormeno, G. I. R., Marques, T., & Barrios, L. (2017). International child abuse prevention: Insights from act raising safe kids. *Child and Adolescent Mental Health, 22*(4), 194-200.
66. Hsu, E. (2003). Parallel group treatment for sexually abused children and their nonoffending parents: An examination of treatment integrity and child and family outcome and satisfaction. [Doctoral dissertation, University of Nebraska-Lincoln]. [*https://digitalcommons.unl.edu/dissertations/AAI3092555/*](https://digitalcommons.unl.edu/dissertations/AAI3092555/)
67. Huey Jr, S. J., Henggeler, S. W., Brondino, M. J., & Pickrel, S. G. (2000). Mechanisms of change in multisystemic therapy: Reducing delinquent behavior through therapist adherence and improved family and peer functioning. *Journal of Consulting and Clinical Psychology, 68*(3), 451.
68. Hukkelberg, S. S., & Ogden, T. (2013). Working alliance and treatment fidelity as predictors of externalizing problem behaviors in parent management training. *Journal of Consulting and Clinical Psychology, 81*(6), 1010.
69. Hutchings, J., Bywater, T., Daley, D., Gardner, F., Whitaker, C., Jones, K., Eames, C., & Edwards, R. T. (2007). Parenting intervention in sure start services for children at risk of developing conduct disorder: Pragmatic randomised controlled trial. *BMJ, 334*(7595), 678.
70. Kjøbli, J., Bjørknes, R., & Askeland, E. (2012). Adherence to brief parent training as a predictor of parent and child outcomes in real‐world settings. *Journal of Children's Services*, *7*(3), 165-177.
71. Kogan, S. M., Lei, M.-K., Brody, G. H., Futris, T. G., Sperr, M., & Anderson, T. (2016). Implementing family-centered prevention in rural African American communities: A randomized effectiveness trial of the strong African American families program. *Prevention Science, 17*(2), 248-258.
72. Kolko, D. J., Baumann, B. L., Herschell, A. D., Hart, J. A., Holden, E. A., & Wisniewski, S. R. (2012). Implementation of AF-CBT by community practitioners serving child welfare and mental health: A randomized trial. *Child Maltreatment, 17*(1), 32-46.
73. Kumpfer, K. L., Scheier, L. M., & Brown, J. (2018). Strategies to avoid replication failure with evidence-based prevention interventions: Case examples from the Strengthening Families Program. *Evaluation & the Health Professions*, *43*(2), 75-89.
74. Kumpfer, K. L., Xie, J., & O’Driscoll, R. (2012). Effectiveness of a culturally adapted Strengthening Families Program 12–16 years for high-risk Irish families. *Child & Youth Care Forum*, *41*(2), 173-195.
75. Lachman, J. M. (2016). Building a rondavel of support: The development and pilot randomised controlled trial of a parenting programme to reduce the risk of child maltreatment in low-income families with children aged three to eight years in south Africa. . [Doctoral dissertation, University of Oxford]. <https://ethos.bl.uk/OrderDetails.do?uin=uk.bl.ethos.724973>
76. Lachman, J. M., Alampay, L., Alinea, C., Gardner, F., Hutchings, J., Ward, C., & Madrid, B. (2018). Parenting for lifelong health (PLH) - Masayang Pamilya (MAPA) evaluation study (PLH-MAPA). *Clinical Trials Protocol Registration and Results System*. <https://clinicaltrials.gov/ct2/show/NCT03205449/>
77. Lachman, J. M., Kelly, J., Cluver, L., Ward, C. L., Hutchings, J., & Gardner, F. (2018). Process evaluation of a parenting program for low-income families in south africa. *Research on Social Work Practice, 28*(2), 188-202.
78. Lange, A. M., Scholte, R. H., van Geffen, W., Timman, R., Busschbach, J. J., & van der Rijken, R. E. (2015). The lack of cross-national equivalence of a therapist adherence measure (TAM-R) in multisystemic therapy (MST). *European Journal of Psychological Assessment*, *32*(4), 312-325.
79. Lange, A. M., van der Rijken, R. E., Busschbach, J. J., Delsing, M. J., & Scholte, R. H. (2017). It’s not just the therapist: Therapist and country-wide experience predict therapist adherence and adolescent outcome. *Child & Youth Care Forum, 46*(4), 455-471.
80. Lange, A. M., van der Rijken, R. E., Delsing, M. J., Busschbach, J. J., & Scholte, R. H. (2019). Development of therapist adherence in relation to treatment outcomes of adolescents with behavioral problems. *Journal of Clinical Child and Adolescent Psychology, 48*(sup1), S337-S346.
81. Lange, A. M., van der Rijken, R. E., Delsing, M. J., Busschbach, J. J., van Horn, J. E., & Scholte, R. H. (2017). Alliance and adherence in a systemic therapy. *Child and Adolescent Mental Health, 22*(3), 148-154.
82. Lebensohn‐Chialvo, F., Rohrbaugh, M. J., & Hasler, B. P. (2019). Fidelity failures in brief strategic family therapy for adolescent drug abuse: A clinical analysis. *Family Process, 58*(2), 305-317.
83. Leer, J., & Lopez-Boo, F. (2019). Assessing the quality of home visit parenting programs in Latin America and the Caribbean. *Early Child Development and Care, 189*(13), 2183-2196.
84. Leonard, A. M., & Jenelle, R. (2014). Evaluating how providers’ competency to deliver SafeCare® relates to provider training and family outcomes. *.* [Doctoral dissertation, Georgia State University]. <https://scholarworks.gsu.edu/cgi/viewcontent.cgi?article=1017&context=psych_hontheses>
85. Lester, S. N. (2015). Evaluation of the parent centre's positive parenting skills training programme: A randomised controlled trial. [Doctoral dissertation, University of Cape Town]. <https://open.uct.ac.za/handle/11427/15615>.
86. Lieberman, A. F., Van Horn, P., & Ippen, C. G. (2005). Toward evidence-based treatment: Child-parent psychotherapy with preschoolers exposed to marital violence. *Journal of the American Academy of Child & Adolescent Psychiatry, 44*(12), 1241-1248.
87. Lindsay, G., Strand, S., & Davis, H. (2011). A comparison of the effectiveness of three parenting programmes in improving parenting skills, parent mental-well being and children's behaviour when implemented on a large scale in community settings in 18 english local authorities: The parenting early intervention pathfinder (PEIP). *BMC Public Health, 11*(1), 962.
88. Löfholm, C. A., Eichas, K., & Sundell, K. (2014). The swedish implementation of multisystemic therapy for adolescents: Does treatment experience predict treatment adherence? *Journal of Clinical Child & Adolescent Psychology, 43*(4), 643-655.
89. Lyon, A. R., & Budd, K. S. (2010). A community mental health implementation of parent–child interaction therapy (PCIT). *Journal of Child and Family Studies, 19*(5), 654-668.
90. Maaskant, A. M., van Rooij, F. B., Overbeek, G. J., Oort, F. J., & Hermanns, J. M. (2016). Parent training in foster families with children with behavior problems: Follow-up results from a randomized controlled trial. *Children and Youth Services Review, 70*, 84-94.
91. MacPherson, H. A., Mackinaw-Koons, B., Leffler, J. M., & Fristad, M. A. (2016). Pilot effectiveness evaluation of community-based multi-family psychoeducational psychotherapy for childhood mood disorders. *Couple and Family Psychology: Research and Practice, 5*(1), 43.
92. Maddox, K. C. (2011). Family transitions: A pilot study evaluation of a six-week support group for families going through divorce. [Doctoral dissertation, Sam Houston State University]. <https://search.proquest.com/openview/848ec2169bd8084aea4d40cf6b159d7d/1?pq-origsite=gscholar&cbl=18750&diss=y&casa_token=ck6lJKg4CpkAAAAA:h0P8GxT6NFPVmth5A2n45XZW9CJ_MdYvE06c5Vr7eC7RdcPcMDKU859t6C41ZErFEq3VjaZmhLo>
93. McKenzie, R., Dallos, R., Stedmon, J., Hancocks, H., Vickery, P. J., Ewings, P., Barton, A., Vassallo, T., & Myhill, C. (2019). Safe, a new therapeutic intervention for families of children with autism: Study protocol for a feasibility randomised controlled trial. *BMJ open, 9*(5), e025006.
94. Mejia, A., Emsley, R., Fichera, E., Maalouf, W., Segrott, J., & Calam, R. (2018). Protecting adolescents in low-and middle-income countries from interpersonal violence (pro youth trial): Study protocol for a cluster randomized controlled trial of the Strengthening Families Programme 10-14 (“Familias Fuertes”) in Panama. *Trials, 19*(1), 320.
95. Morpeth, L., Blower, S., Tobin, K., Taylor, R. S., Bywater, T., Edwards, R. T., Axford, N., Lehtonen, M., Jones, C., & Berry, V. (2017). The effectiveness of the incredible years pre-school parenting programme in the United Kingdom: A pragmatic randomised controlled trial. *Child Care in Practice, 23*(2), 141-161.
96. Multisite Violence Prevention Project. (2014). Implementation and process effects on prevention outcomes for middle school students. *Journal of Clinical Child and Adolescent Psychology, 43*(3), 743-485.
97. Murray, D. W., Lawrence, J. R., & LaForett, D. R. (2018). The Incredible Years® programs for adhd in young children: A critical review of the evidence. *Journal of Emotional and Behavioral Disorders, 26*(4), 195-208.
98. O’Brien, R., Buston, K., Wight, D., McGee, E., White, J., & Henderson, M. (2019). A realist process evaluation of enhanced triple p for baby and mellow bumps, within a trial of healthy relationship initiatives for the very early years (THRIVE): Study protocol for a randomized controlled trial. *Trials, 20*(1), 351.
99. Oxford, M. L., Spieker, S. J., Lohr, M. J., Fleming, C. B., Dillon, C., & Rees, J. (2018). Ensuring implementation fidelity of a 10-week home visiting program in two randomized clinical trials. *Maternal and Child Health Journal, 22*(3), 376-383.
100. Palmer, M., San José Cáceres, A., Tarver, J., Howlin, P., Slonims, V., Pellicano, E., & Charman, T. (2020). Feasibility study of the national autistic society EarlyBird parent support programme. *Autism, 24*(1), 147-159.
101. Palmer, R. (2012). Assessing the relationship between SafeCare fidelity and competence measures. [Doctoral dissertation, Georgia State University]*.* https://scholarworks.gsu.edu/cgi/viewcontent.cgi?article=1238&context=iph_theses
102. Phan, T. V. (2018). Understanding poor treatment outcomes despite therapist adherence in multisystemic therapy. [Doctoral dissertation, Alliant International University]. <https://search.proquest.com/docview/2167289691?pq-origsite=gscholar&fromopenview=true>
103. Pierce, J. P., James, L. E., Messer, K., Myers, M. G., Williams, R. E., & Trinidad, D. R. (2008). Telephone counseling to implement best parenting practices to prevent adolescent problem behaviors. *Contemporary Clinical Trials, 29*(3), 324-334.
104. Puffer, E. S., Friis-Healy, E. A., Giusto, A., Stafford, S., & Ayuku, D. (2019). Development and implementation of a family therapy intervention in kenya: A community-embedded lay provider model. *Global Social Welfare*, 1-18.
105. Reid, J. B., Eddy, J. M., Fetrow, R. A., & Stoolmiller, M. (1999). Description and immediate impacts of a preventive intervention for conduct problems. *American Journal of Community Psychology, 27*(4), 483-518.
106. Rendu, A. (2004). Treatment adherence in a behaviourally based parenting program. [Doctoral dissertation, University College London]. <https://discovery.ucl.ac.uk/id/eprint/10097909/1/Treatment_adherence_in_a_behav.pdf>
107. Robbins, M. S., Feaster, D. J., Horigian, V. E., Puccinelli, M. J., Henderson, C., & Szapocznik, J. (2011). Therapist adherence in brief strategic family therapy for adolescent drug abusers. *Journal of Consulting and Clinical Psychology, 79*(1), 43.
108. Rodrigo, M. J., Martín, J. C., Mateos, A., Pastor, C., & Guerra, M. (2013). Impact of the" learning together, growing in family" programme on the professionals and attention to families services. *The Central and Eastern European Online Library.*
109. Rogers, S. J., & Vismara, L. (2014). Interventions for infants and toddlers at risk for autism spectrum disorder. *Handbook of Autism and Pervasive Developmental Disorders, Fourth Edition*.
110. Roggman, L. A., Boyce, L. K., Cook, G. A., & Jump, V. K. (2001). Inside home visits: A collaborative look at process and quality. *Early Childhood Research Quarterly, 16*(1), 53-71.
111. Roggman, L. A., Cook, G. A., Innocenti, M. S., Jump Norman, V., Boyce, L. K., Christiansen, K., & Peterson, C. A. (2016). Home visit quality variations in two early head start programs in relation to parenting and child vocabulary outcomes. *Infant Mental Health Journal, 37*(3), 193-207.
112. Ronan, K., Davies, G., Wikman, R., Canoy, D., Jarret, M., & Evans, C. (2016). Family-centered, feedback-informed therapy for conduct disorder: Findings from an empirical case study. *Couple and Family Psychology: Research and Practice*, *5*(3), 137.
113. Ryan, S. R., Cunningham, P. B., Foster, S. L., Brennan, P. A., Brock, R. L., & Whitmore, E. (2013). Predictors of therapist adherence and emotional bond in multisystemic therapy: Testing ethnicity as a moderator. *Journal of Child and Family Studies, 22*(1), 122-136.
114. Saïas, T., Lerner, E., Greacen, T., Simon-Vernier, E., Emer, A., Pintaux, E., Guédeney, A., Dugravier, R., Tereno, S., & Falissard, B. (2012). Evaluating fidelity in home-visiting programs a qualitative analysis of 1058 home visit case notes from 105 families. *PLoS One, 7*(5).
115. Satterfield, L. W. (2013). The effectiveness of functional family therapy in an Irish context: An examination of international implementation. [Indiana University]
116. Schoenwald, S. K., Carter, R. E., Chapman, J. E., & Sheidow, A. J. (2008). Therapist adherence and organizational effects on change in youth behavior problems one year after multisystemic therapy. *Administration and Policy in Mental Health and Mental Health Services Research, 35*(5), 379.
117. Schoenwald, S. K., Chapman, J. E., Sheidow, A. J., & Carter, R. E. (2009). Long-term youth criminal outcomes in mst transport: The impact of therapist adherence and organizational climate and structure. *Journal of Clinical Child and Adolescent Psychology, 38*(1), 91-105.
118. Schoenwald, S. K., Halliday-Boykins, C., & Henggeler, S. W. (2003). Client‐level predictors of adherence to MST in community service settings. *Family Process, 42*(3), 345-359.
119. Schoenwald, S. K., Henggeler, S. W., Brondino, M. J., & Rowland, M. D. (2000). Multisystemic therapy: Monitoring treatment fidelity. *Family Process, 39*(1), 83-103.
120. Schoenwald, S. K., Letourneau, E. J., & Halliday-Boykins, C. (2005). Predicting therapist adherence to a transported family-based treatment for youth. *Journal of Clinical Child and Adolescent Psychology, 34*(4), 658-670.
121. Schoenwald, S. K., Sheidow, A. J., & Chapman, J. E. (2009). Clinical supervision in treatment transport: Effects on adherence and outcomes. *Journal of Consulting and Clinical Psychology, 77*(3), 410.
122. Schoenwald, S. K., Sheidow, A. J., & Letourneau, E. J. (2004). Toward effective quality assurance in evidence-based practice: Links between expert consultation, therapist fidelity, and child outcomes. *Journal of Clinical Child and Adolescent Psychology, 33*(1), 94-104.
123. Scott, S., Carby, A., & Rendu, A. (2008). Impact of therapists’ skill on effectiveness of parenting groups for child antisocial behavior. *Institute of Psychiatry, Kings College London*.
124. Scott, S., Sylva, K., Doolan, M., Price, J., Jacobs, B., Crook, C., & Landau, S. (2010). Randomised controlled trial of parent groups for child antisocial behaviour targeting multiple risk factors: The spokes project. *Journal of Child Psychology and Psychiatry, 51*(1), 48-57.
125. Sexton, T., & Turner, C. W. (2010). The effectiveness of functional family therapy for youth with behavioral problems in a community practice setting. *Journal of Family Psychology, 24*(3), 339.
126. Shenderovich, Y., Eisner, M., Cluver, L., Doubt, J., Berezin, M., Majokweni, S., & Murray, A. L. (2019). Delivering a parenting program in south africa: The impact of implementation on outcomes. *Journal of Child and Family Studies, 28*(4), 1005-1017.
127. Sheshko, D. M., Lee, C. M., & Gagné, M.-H. (2020, March 12). Multimethod Adherence Measurement in an Evidence-Based Parenting Program. Practice Innovations. Advance online publication. <http://dx.doi.org/10.1037/pri0000110>.
128. Sigmarsdóttir, M., Forgatch, M. S., Guðmundsdóttir, E. V., Thorlacius, Ö., Svendsen, G. T., Tjaden, J., & Gewirtz, A. H. (2019). Implementing an evidence-based intervention for children in europe: Evaluating the full-transfer approach. *Journal of Clinical Child and Adolescent Psychology, 48*(sup1), S312-S325.
129. Sigmarsdóttir, M., & Guðmundsdóttir, E. V. (2013). Implementation of parent management training—Oregon model (PMTO) in Iceland: Building sustained fidelity. *Family Process, 52*(2), 216-227.
130. Simkiss, D. E., Snooks, H. A., Stallard, N., Davies, S., Thomas, M. A., Anthony, B., Winstanley, S., Wilson, L., & Stewart-Brown, S. (2010). Measuring the impact and costs of a universal group based parenting programme: Protocol and implementation of a trial. *BMC Public Health, 10*(1), 364.
131. Singer, A. J. (2001). Therapist and observer ratings of therapist fidelity to a family-based prevention model. [Doctoral dissertation, Fordham University, Ann Arbor]. https://research.library.fordham.edu/dissertations/AAI3022799/
132. Smith, J. D., Dishion, T. J., Brown, K., Ramos, K., Knoble, N. B., Shaw, D. S., & Wilson, M. N. (2016). An experimental study of procedures to enhance ratings of fidelity to an evidence-based family intervention. *Prevention Science, 17*(1), 62-70.
133. Smith, J. D., Dishion, T. J., Shaw, D. S., & Wilson, M. N. (2013). Indirect effects of fidelity to the family check-up on changes in parenting and early childhood problem behaviors. *Journal of Consulting and Clinical Psychology, 81*(6), 962.
134. Smith, J. D., Rudo-Stern, J., Dishion, T. J., Stormshak, E. A., Montag, S., Brown, K., Ramos, K., Shaw, D. S., & Wilson, M. N. (2019). Effectiveness and efficiency of observationally assessing fidelity to a family-centered child intervention: A quasi-experimental study. *Journal of Clinical Child and Adolescent Psychology, 48*(1), 16-28.
135. Smith, J. D., Stormshak, E. A., & Kavanagh, K. (2015). Results of a pragmatic effectiveness–implementation hybrid trial of the family check-up in community mental health agencies. *Administration and Policy in Mental Health and Mental Health Services Research, 42*(3), 265-278.
136. Snider, M. D. (2019). Examining the impact of treatment fidelity on client outcomes in a statewide implementation of parent-child interaction therapy. [Doctoral dissertation, West Virginia University]. <https://researchrepository.wvu.edu/cgi/viewcontent.cgi?article=4793&context=etd>.
137. Solomon, R., Van Egeren, L. A., Mahoney, G., Huber, M. S. Q., & Zimmerman, P. (2014). Play project home consultation intervention program for young children with autism spectrum disorders: A randomized controlled trial. *Journal of Developmental and Behavioral Pediatrics, 35*(8), 475.
138. St. George, S. M., Huang, S., Vidot, D. C., Smith, J. D., Brown, C. H., & Prado, G. (2016). Factors associated with the implementation of the Familias Unidas intervention in a type 3 translational trial. *Translational behavioral medicine, 6*(1), 105-114.
139. St. George, S. M., Wilson, D. K., McDaniel, T., & Alia, K. A. (2016). Process evaluation of the project shine intervention for African American families: An integrated positive parenting and peer monitoring approach to health promotion. *Health Promotion Practice, 17*(4), 557-568.
140. Stern, S. B., Alaggia, R., Watson, K., & Morton, T. R. (2008). Implementing an evidence-based parenting program with adherence in the real world of community practice. *Research on Social Work Practice, 18*(6), 543-554.
141. Sterrett-Hong, E. M., Karam, E., & Kiaer, L. (2017). Statewide implementation of parenting with love and limits among youth with co-existing internalizing and externalizing functional impairments reduces return to service rates and treatment costs. *Administration and Policy in Mental Health and Mental Health Services Research, 44*(5), 792-809.
142. Stokes, J. (2014). Effectiveness of community-delivered parent-child interaction therapy compared to treatment as usual. [Doctoral dissertation, West Virginia University]*.* https://researchrepository.wvu.edu/etd/197/
143. Strauss, K., Vicari, S., Valeri, G., D’Elia, L., Arima, S., & Fava, L. (2012). Parent inclusion in early intensive behavioral intervention: The influence of parental stress, parent treatment fidelity and parent-mediated generalization of behavior targets on child outcomes. *Research in Developmental Disabilities, 33*(2), 688-703.
144. Suchman, N. E., DeCoste, C., Borelli, J. L., & McMahon, T. J. (2018). Does improvement in maternal attachment representations predict greater maternal sensitivity, child attachment security and lower rates of relapse to substance use? A second test of mothering from the inside out treatment mechanisms. *Journal of Substance Abuse Treatment, 85*, 21-30.
145. Suchman, N. E., Decoste, C., Rosenberger, P., & McMahon, T. J. (2012). Attachment‐based intervention for substance‐using mothers: A preliminary test of the proposed mechanisms of change. *Infant Mental Health Journal, 33*(4), 360-371.
146. Suchman, N. E., Ordway, M. R., de las Heras, L., & McMahon, T. J. (2016). Mothering from the inside out: Results of a pilot study testing a mentalization-based therapy for mothers enrolled in mental health services. *Attachment and Human Development, 18*(6), 596-617.
147. Sundell, K., Hansson, K., Löfholm, C. A., Olsson, T., Gustle, L.-H., & Kadesjö, C. (2008). The transportability of multisystemic therapy to sweden: Short-term results from a randomized trial of conduct-disordered youths. *Journal of Family Psychology, 22*(4), 550.
148. Taylor, W. D., Asgary-Eden, V., Lee, C. M., & LaRoche, K. J. (2015). Service providers’ adherence to an evidence-based parenting program: What are they missing and why? *Journal of Child and Family Studies, 24*(1), 50-56.
149. Thijssen, J., Albrecht, G., Muris, P., & de Ruiter, C. (2017). Treatment fidelity during therapist initial training is related to subsequent effectiveness of parent management training—oregon model. *Journal of Child and Family Studies, 26*(7), 1991-1999.
150. Timmer, S. G., Hawk, B., Forte, L. A., Boys, D. K., & Urquiza, A. J. (2019). An open trial of parent–child care (pc-care)-a 6-week dyadic parenting intervention for children with externalizing behavior problems. *Child Psychiatry anf Human Development, 50*(1), 1-12.
151. Tiwari, A. (2010). A comparison of methods to assess practitioner fidelity in a parent-training program. [Doctoral dissertation, Georgia State University]. <https://citeseerx.ist.psu.edu/viewdoc/download?doi=10.1.1.425.6886&rep=rep1&type=pdf>
152. Travis, J. (2012). Examining trainee treatment session fidelity: Impact on the implementation of parent-child interaction therapy (PCIT). [Doctoral dissertation, Auburn University]. <https://etd.auburn.edu/bitstream/handle/10415/3076/Jamie%20Travis%20Thesis.pdf?sequence=2&isAllowed=y>
153. Travis, J. K., & Brestan-Knight, E. (2013). A pilot study examining trainee treatment session fidelity when parent–child interaction therapy (pcit) is implemented in community settings. *The Journal of Behavioral Health Services and Research, 40*(3), 342-354.
154. Webster-Stratton, C. H., Reid, M. J., & Marsenich, L. (2014). Improving therapist fidelity during implementation of evidence-based practices: Incredible years program. *Psychiatric Services, 65*(6), 789-795.
155. Whitaker, D. J., Ryan, K. A., Wild, R. C., Self-Brown, S., Lutzker, J. R., Shanley, J. R., Edwards, A. M., McFry, E. A., Moseley, C. N., & Hodges, A. E. (2012). Initial implementation indicators from a statewide rollout of SafecCare within a child welfare system. *Child Maltreatment, 17*(1), 96-101.
156. Wydra, M. (2013). Does adoption therapy work?: Evaluating a therapy program for adopted children and their families. [Doctoral dissertation, University of Maryland]. <https://drum.lib.umd.edu/handle/1903/14495>.

# Part 2

1. Askeland, E., Forgatch, M. S., Apeland, A., Reer, M., & Grønlie, A. A. (2019). Scaling up an empirically supported intervention with long-term outcomes: The nationwide implementation of Generation PMTO in Norway. *Prevention Science, 20*(8), 1189-1199.
2. Berkel, C., Mauricio, A. M., Sandler, I. N., Wolchik, S. A., Gallo, C. G., & Brown, C. H. (2018). The cascading effects of multiple dimensions of implementation on program outcomes: A test of a theoretical model. *Prevention Science, 19*(6), 782-794.
3. Bustos, C. E. (2011). *Parent experiences of a family-centered intervention: Examining ethnocultural group* differences. [Doctoral dissertation, University of Oregon]. https://scholarsbank.uoregon.edu/xmlui/handle/1794/11228.
4. Byrnes, H. F., Miller, B. A., Aalborg, A. E., Plasencia, A. V., & Keagy, C. D. (2010). Implementation fidelity in adolescent family-based prevention programs: Relationship to family engagement. *Health Education Research, 25*(4), 531-541.
5. Bywater, T., Gridley, N., Berry, V., Blower, S., & Tobin, K. (2019). The parent programme implementation checklist (PPIC): The development and testing of an objective measure of skills and fidelity for the delivery of parent programmes. *Child Care in Practice, 25*(3), 281-309.
6. Chiapa, A., Smith, J. D., Kim, H., Dishion, T. J., Shaw, D. S., & Wilson, M. N. (2015). The trajectory of fidelity in a multiyear trial of the family check-up predicts change in child problem behavior. *Journal of Consulting and Clinical Psychology, 83*(5), 1006.
7. Costello, A. H., Roben, C. K., Schein, S. S., Blake, F., & Dozier, M. (2019). Monitoring provider fidelity of a parenting intervention using observational methods. *Professional Psychology: Research and Practice, 50*(4), 264.
8. Eames, C., Daley, D., Hutchings, J., Hughes, J., Jones, K., Martin, P., & Bywater, T. (2008). The leader observation tool: A process skills treatment fidelity measure for the incredible years parenting programme. *Child: Care, Health and Development, 34*(3), 391-400.
9. Eames, C., Daley, D., Hutchings, J., Whitaker, C., Jones, K., Hughes, J., & Bywater, T. (2009). Treatment fidelity as a predictor of behaviour change in parents attending group‐based parent training. *Child: Care, Health and Development, 35*(5), 603-612.
10. Feely, M., Seay, K. D., Lanier, P., Auslander, W., & Kohl, P. L. (2018). Measuring fidelity in research studies: A field guide to developing a comprehensive fidelity measurement system. *Child and Adolescent Social Work Journal, 35*(2), 139-152.
11. Forgatch, M. S., & DeGarmo, D. S. (2011). Sustaining fidelity following the nationwide PMTO™ implementation in Norway. *Prevention Science, 12*(3), 235-246.
12. Forgatch, M. S., Patterson, G. R., & DeGarmo, D. S. (2005). Evaluating fidelity: Predictive validity for a measure of competent adherence to the Oregon model of parent management training. *Behavior Therapy, 36*(1), 3-13.
13. Giannotta, F., Özdemir, M., & Stattin, H. (2019). The implementation integrity of parenting programs: Which aspects are most important? *Child and Youth Care Forum*, *48*, 917-933.
14. Gross, T., Alex Mason, W., Parra, G., Oats, R., Ringle, J. L., & Haggerty, K. (2015). Adherence and dosage contributions to parenting program quality. *Journal of the Society for Social Work and Research, 6*(4), 467-489.
15. Hill, L. G., & Owens, R. W. (2013). Component analysis of adherence in a family intervention. *Health Education*, *113*(4), 264-280.
16. Hogue, A., Dauber, S., Chinchilla, P., Fried, A., Henderson, C., Inclan, J., Reiner, R. H., & Liddle, H. A. (2008). Assessing fidelity in individual and family therapy for adolescent substance abuse. *Journal of Substance Abuse Treatment, 35*(2), 137-147.
17. Hogue, A., Liddle, H. A., Rowe, C., Turner, R. M., Dakof, G. A., & LaPann, K. (1998). Treatment adherence and differentiation in individual versus family therapy for adolescent substance abuse. *Journal of Counseling Psychology, 45*(1), 104.
18. Hogue, A., Liddle, H. A., Singer, A., & Leckrone, J. (2005). Intervention fidelity in family‐based prevention counseling for adolescent problem behaviors. *Journal of Community Psychology, 33*(2), 191-211.
19. Hukkelberg, S. S., & Ogden, T. (2013). Working alliance and treatment fidelity as predictors of externalizing problem behaviors in parent management training. *Journal of Consulting and Clinical Psychology, 81*(6), 1010.
20. Kogan, S. M., Lei, M.-K., Brody, G. H., Futris, T. G., Sperr, M., & Anderson, T. (2016). Implementing family-centered prevention in rural African American communities: A randomized effectiveness trial of the strong African American families program. *Prevention Science, 17*(2), 248-258.
21. Leer, J., & Lopez-Boo, F. (2019). Assessing the quality of home visit parenting programs in Latin America and the Caribbean. *Early Child Development and Care, 189*(13), 2183-2196.
22. Rendu, A. (2004). Treatment adherence in a behaviourally based parenting program. [Doctoral dissertation, University College London]. <https://discovery.ucl.ac.uk/id/eprint/10097909/1/Treatment_adherence_in_a_behav.pdf>
23. Roggman, L. A., Boyce, L. K., Cook, G. A., & Jump, V. K. (2001). Inside home visits: A collaborative look at process and quality. *Early Childhood Research Quarterly, 16*(1), 53-71.
24. Roggman, L. A., Cook, G. A., Innocenti, M. S., Jump Norman, V., Boyce, L. K., Christiansen, K., & Peterson, C. A. (2016). Home visit quality variations in two early head start programs in relation to parenting and child vocabulary outcomes. *Infant Mental Health Journal, 37*(3), 193-207.
25. Scott, S., Carby, A., & Rendu, A. (2008). Impact of therapists’ skill on effectiveness of parenting groups for child antisocial behavior. [Institute of Psychiatry, Kings College London.] <https://www.incredibleyears.com/wp-content/uploads/therapists-skill_08.pdf>.
26. Shenderovich, Y., Eisner, M., Cluver, L., Doubt, J., Berezin, M., Majokweni, S., & Murray, A. L. (2019). Delivering a parenting program in south africa: The impact of implementation on outcomes. *Journal of Child and Family Studies, 28*(4), 1005-1017.
27. Sigmarsdóttir, M., Forgatch, M. S., Guðmundsdóttir, E. V., Thorlacius, Ö., Svendsen, G. T., Tjaden, J., & Gewirtz, A. H. (2019). Implementing an evidence-based intervention for children in europe: Evaluating the full-transfer approach. *Journal of Clinical Child and Adolescent Psychology, 48*(sup1), S312-S325.
28. Sigmarsdóttir, M., & Guðmundsdóttir, E. V. (2013). Implementation of parent management training—oregon model (PMTO) in Iceland: Building sustained fidelity. *Family Process, 52*(2), 216-227.
29. Singer, A. J. (2001). Therapist and observer ratings of therapist fidelity to a family-based prevention model. [Doctoral dissertation, Fordham University, Ann Arbor]. https://research.library.fordham.edu/dissertations/AAI3022799/
30. Smith, J. D., Dishion, T. J., Brown, K., Ramos, K., Knoble, N. B., Shaw, D. S., & Wilson, M. N. (2016). An experimental study of procedures to enhance ratings of fidelity to an evidence-based family intervention. *Prevention Science, 17*(1), 62-70.
31. Smith, J. D., Dishion, T. J., Shaw, D. S., & Wilson, M. N. (2013). Indirect effects of fidelity to the Family Check-Up on changes in parenting and early childhood problem behaviors. *Journal of Consulting and Clinical Psychology, 81*(6), 962.
32. Smith, J. D., Rudo-Stern, J., Dishion, T. J., Stormshak, E. A., Montag, S., Brown, K., Ramos, K., Shaw, D. S., & Wilson, M. N. (2019). Effectiveness and efficiency of observationally assessing fidelity to a family-centered child intervention: A quasi-experimental study. *Journal of Clinical Child and Adolescent Psychology, 48*(1), 16-28.
33. Smith, J. D., Stormshak, E. A., & Kavanagh, K. (2015). Results of a pragmatic effectiveness–implementation hybrid trial of the family check-up in community mental health agencies. *Administration and Policy in Mental Health and Mental Health Services Research, 42*(3), 265-278.
34. Snider, M. D. (2019). Examining the impact of treatment fidelity on client outcomes in a statewide implementation of parent-child interaction therapy. [Doctoral dissertation, West Virginia University]. <https://researchrepository.wvu.edu/cgi/viewcontent.cgi?article=4793&context=etd>.
35. Solomon, R., Van Egeren, L. A., Mahoney, G., Huber, M. S. Q., & Zimmerman, P. (2014). Play project home consultation intervention program for young children with autism spectrum disorders: A randomized controlled trial. *Journal of Developmental and Behavioral Pediatrics, 35*(8), 475.
36. St. George, S. M., Huang, S., Vidot, D. C., Smith, J. D., Brown, C. H., & Prado, G. (2016). Factors associated with the implementation of the Familias Unidas intervention in a type 3 translational trial. *Translational Behavioral Medicine, 6*(1), 105-114.
37. Sterrett-Hong, E. M., Karam, E., & Kiaer, L. (2017). Statewide implementation of parenting with love and limits among youth with co-existing internalizing and externalizing functional impairments reduces return to service rates and treatment costs. *Administration and Policy in Mental Health and Mental Health Services Research, 44*(5), 792-809.
38. Strauss, K., Vicari, S., Valeri, G., D’Elia, L., Arima, S., & Fava, L. (2012). Parent inclusion in early intensive behavioral intervention: The influence of parental stress, parent treatment fidelity and parent-mediated generalization of behavior targets on child outcomes. *Research in Developmental Disabilities, 33*(2), 688-703.
39. Timmer, S. G., Hawk, B., Forte, L. A., Boys, D. K., & Urquiza, A. J. (2019). An open trial of parent–child care (pc-care)-a 6-week dyadic parenting intervention for children with externalizing behavior problems. *Child Psychiatry and Human Development, 50*(1), 1-12.
40. Travis, J. (2012). Examining trainee treatment session fidelity: Impact on the implementation of parent-child interaction therapy (PCIT). [Doctoral dissertation, Auburn University]. <https://etd.auburn.edu/bitstream/handle/10415/3076/Jamie%20Travis%20Thesis.pdf?sequence=2&isAllowed=y>
41. Webster-Stratton, C. H., Reid, M. J., & Marsenich, L. (2014). Improving therapist fidelity during implementation of evidence-based practices: Incredible years program. *Psychiatric Services, 65*(6), 789-795.
