# Supplementary material for: Measures of Facilitator Competent Adherence Used in Parenting Programs and Their Psychometric Properties: A Systematic Review
Source: Clin Child Fam Psychol Rev. 2021 May 21;24(4):834–53. doi: 10.1007/s10567-021-00350-8 (PMC8541983; doi:10.1007/s10567-021-00350-8)
Supplement: Supplementary file 2 — Supplementary file2 (DOCX 27 KB) [file 10567_2021_350_MOESM2_ESM.docx]

**Measures of Facilitator Competent Adherence used in Parenting Programs and their Psychometric Properties: A Systematic Review**

Mackenzie Martin, Bridget Steele, Jamie M. Lachman, and Frances Gardner

Department of Social Policy and Intervention

University of Oxford, United Kingdom

Correspondence regarding this article should be addressed to Mackenzie Martin, Department of Social Policy and Intervention, University of Oxford

Email: Mackenzie.Martin@spi.ox.ac.uk

## Data Screening and Extraction Forms

## Part 1 Title and Abstract Screening Form

**Instructions:** Only continue to the next question if the previous question is answered “yes”. If any question is answered “no”, the study should be excluded from the review.

| **Component** | **Eligibility Criteria** | **Criteria Satisfied?** |
| --- | --- | --- |
| Intervention | Does this study report on a parenting intervention? | - Yes - No |
|  | Does this study report on at least one of the following:  (a) reduced child maltreatment, harsh or dysfunctional parenting, or child conduct problems and/or  (b) improved positive child behaviour management strategies, parent-child bonding/attachment and relationships, or early child development outcomes?  AND  Not focus narrowly on specific child risks, such as poisoning, accidents, and skills training for specific medical conditions or physical disabilities? | - Yes - No |
| Facilitator Competence and/or Adherence | Does this study report make reference to facilitator competence (i.e., quality) and/or adherence (i.e., fidelity)? | - Yes - No |
| - INCLUDE - EXCLUDE | | |

## Part 1 Full-Text Screening Form

**Instructions:** Only continue to the next question if the previous question is answered “yes”. If any question is answered “no”, the study should be excluded from the review.

| **Component** | **Eligibility Criteria** | **Criteria Satisfied?** |
| --- | --- | --- |
| Intervention | Does this study report on a parenting intervention? | - Yes - No |
|  | Does this study report on at least one of the following outcomes:  (a) reduced child maltreatment, harsh or dysfunctional parenting, or child conduct problems and/or  (b) improved positive child behaviour management strategies, parent-child bonding/attachment and relationships, or early child development outcomes?  AND  Not focus narrowly on specific child risks, such as poisoning, accidents, and skills training for specific medical conditions or physical disabilities? | - Yes - No |
|  | Is at least 50% of the programme delivered to parents? | - Yes - No |
|  | Are parents aged 18 years or older and their children aged 0-17? | - Yes - No |
| Facilitator Competence and/or Adherence | Does this study report on a measure of:   1. Facilitator competence/quality, 2. Facilitator adherence/fidelity, 3. Facilitator competent adherence? | - Yes - No |
| - INCLUDE - EXCLUDE | | |

## Part 1 Data Extraction Form

| **General Information** | |
| --- | --- |
| Initials of data extractor |  |
| Date of data extraction (DD-MM-YEAR) |  |
| First author last name |  |
| Study publication year |  |
| **Intervention Characteristics** | |
| Programme title/brand |  |
| Programme objectives |  |
| Programme primary outcomes |  |
| Programme secondary outcomes |  |
| Parent age range |  |
| Child age range |  |
| Country of study |  |
| **Facilitator Characteristics** | |
| Facilitator age range |  |
| Percentage female |  |
| Percentage parents/caregivers |  |
| Experience delivering parenting programs |  |
| Sample size |  |
| Educational background |  |
| Years of experience |  |
| Other |  |
| **Measure** (repeat this section again if the article reports on more than one measure) | |
| Name of measure |  |
| Description of measure |  |
| Domain of measure | - Competence - Adherence - Competent adherence - Competence and adherence - Other |
| Other domain of measure notes |  |
| Assessors of measure | - Supervisor - Facilitator - Parent - Peer - Third party - Researcher - Not specified - Other |
| Other assessor of measure notes |  |
| Format of measure | - Paper - Electronic - Other |
| Other format of measure |  |
| Method of completion | - Memory - Audio - Video - Live - Other: |
| Other method of completion and notes |  |
| Number of items |  |
| Number of subscales |  |
| Number of response options (e.g., 1-7) |  |
| Range of possible scores |  |
| Completion time (minutes) |  |
| Cost (GBP) |  |
| Availability (website link) |  |
| Was coder training required? | - Yes - No |
| If so, number of training hours |  |
| Measure result |  |
