# Supplementary material for: Measures of Facilitator Competent Adherence Used in Parenting Programs and Their Psychometric Properties: A Systematic Review
Source: Clin Child Fam Psychol Rev. 2021 May 21;24(4):834–53. doi: 10.1007/s10567-021-00350-8 (PMC8541983; doi:10.1007/s10567-021-00350-8)
Supplement: Supplementary file 3 — Supplementary file3 (DOCX 31 KB) [file 10567_2021_350_MOESM3_ESM.docx]

**Measures of Facilitator Competent Adherence used in Parenting Programs and their Psychometric Properties: A Systematic Review**

Mackenzie Martin, Bridget Steele, Jamie M. Lachman, and Frances Gardner

Department of Social Policy and Intervention

University of Oxford, United Kingdom

Correspondence regarding this article should be addressed to Mackenzie Martin, Department of Social Policy and Intervention, University of Oxford

Email: Mackenzie.Martin@spi.ox.ac.uk

**Risk of Bias and Quality Assessment Checklists**

## Part 2 Measure Risk of Bias and Quality Checklist

| **Measurement Property** | **Definition** | **Rating** | **Criteria** | **Source** |
| --- | --- | --- | --- | --- |
| Internal consistency (using classic test theory) | Degree of interrelatedness among items | **+** | Cronbach alpha(s) > 0.70 | Terwee et al., 2007 |
|  |  | **?** | Cronbach’s alpha(s) not determined OR dimensionality unknown OR conflicting evidence |  |
|  |  | **-** | Cronbach alpha(s) < 0.70 |  |
| Internal consistency (using item response theory) |  | **+** | Person separation index  > 0.70 | Heinl et al., 2016 |
|  |  | **?** | Person separation index not determined |  |
|  |  | **-** | Person separation index  < 0.70 |  |
| Reliability | Inter-rater assesses scores from different people at the same time, whilst intra-rater assess scores from the same person at different times  Test-retest measures stability over time | **+** | ICC/weighted Kappa  > 0.70  OR  Pearson’s r > 0.80 | Heinl et al., 2016 |
|  |  | **?** | Neither ICC/weighted Kappa or Pearson’s r calculated |  |
|  |  | **-** | ICC/weighted Kappa  > 0.70  OR  Pearson’s r < 0.80 |  |
| Content Validity | The degree to which a measure is an adequate reflection of the construct that it intends to measure | **+** | All items are considered to be relevant for the construct to be measured, for the target population, and for the purpose of the measurement AND the questionnaire is considered to be comprehensive | Terwee et al., 2007 |
|  |  | **?** | Not enough information available |  |
|  |  | **-** | Not all items are considered to be relevant for the construct to be measured, for the target population, and for the purpose of the measurement OR the questionnaire is not considered to be comprehensive |  |
| Construct validity (classic test theory methods) | Degree to which scores of a measure are an adequate reflection of the dimensionality of the construct to be measured | **+** | Factors should explain at least 50% of the variance  OR  CFI or TLI or comparable measure > 0.95 AND (RMSEA<0.06 OR SRMR <0.08) | Terwee et al., 2007 and COSMIN guidelines |
|  |  | **?** | Not all information for ‘+’ is reported |  |
|  |  | **-** | Criteria for ‘+’ not met |  |
| Construct validity (item response theory methods) |  | **+** | Residual correlations among the items after controlling for the dominant factor < 0.20  OR  Q3’s <0/37, item scalability > 0.30, IRT model fit: G2>0.01, no DIF for important subject characteristics (such as gender, age, education): McFadden’s R2<0/02  OR  No non-uniform DIF | Terwee et al., 2007 and COSMIN guidelines |
|  |  | **?** | Important statistics not reported |  |
|  |  | **-** | Criteria for ‘+’ not met |  |
| Convergent  / divergent validity | The degree to which relationships between scores on one measure are sufficiently related (convergent) or unrelated (divergent) to scores on other instruments measuring similar or dissimilar constructs | **+** | Correlations with instruments measuring the same construct > 0.50  OR  At least 75% of the results are in accordance with the hypotheses AND correlation with related constructs is higher than with unrelated constructs | Terwee et al., 2007 |
|  |  | **?** | Solely correlations determined with unrelated constructs |  |
|  |  | **-** | Correlations with instruments measuring the same constructs <0.50  OR  75% of the results are in accordance with the hypotheses OR correlation with related constructs is higher than with unrelated constructs |  |
| Criterion Validity | Degree to which scores of a measure are an adequate reflection of the gold standard | **+** | Convincing arguments that gold standard is “gold”  AND  Correlation with gold standard > 0.70 OR (sensitivity AND specificity > 70%) |  |
|  |  | **?** | Not all information for ‘+’ reported |  |
|  |  | **-** | Criteria for ‘+’ not met |  |

## Part 2 Study Risk of Bias and Quality Checklist

| Session Sampling Method | The method through which programme sessions for assessment were selected for observation | + | All sessions were observed, or random sampling was used | Walton et al., 2017 |
| --- | --- | --- | --- | --- |
|  |  | ? | No information was provided about the session sampling method used |  |
|  |  | - | Other sampling methods, such as purposive, opportunity, or stratified, were used |  |
| Assessors | The number and independence of assessors | + | Two or more independent assessors were used | Walton et al., 2017 |
|  |  | ? | No information was provided about the number or independence of assessors |  |
|  |  | - | Only one assessor was used |  |
| Facilitator Reactivity | The degree to which measures were taken to reduce facilitator reactivity to observation | + | Some information was provided on steps taken to reduce reactivity | Gardner, 2000 |
|  |  | ? | No information was provided on facilitator reactivity |  |
|  |  | - | Information was provided suggesting that inadequate steps were taken to reduce reactivity |  |

## Part 2 Measure Practicality Checklist

| Feasibility and Sustainability of Assessor Training | Whether the amount of training provided to assessors was reasonable and feasible for future programme delivery | **+** | Yes, the amount of training provided to assessors seems reasonable and sustainable | Milne & Reiser, 2012 |
| --- | --- | --- | --- | --- |
|  |  | **?** | No information was provided about assessor training or not enough information was provided to make an assessment |  |
|  |  | **-** | No, the amount of training provided to assessors does not seem reasonable and sustainable |  |
| Measure Utility | The extent to which the measure appears useable in practice | **+** | The measure seems easy to use and applicable to practice | Barkham et al., 1998; Milne & Reiser, 2012 |
|  |  | **?** | Not enough information was provided to make an assessment |  |
|  |  | **-** | The measure seems difficult and impractical to use |  |
| Measure Availability | Whether the measure is published online so that other researchers may access it | **+** | The measure was reported in the study or it was easily found through a quick internet search | Barkham et al., 1998; Milne & Reiser, 2012 |
|  |  | **-** | The measure was not included in the study and it was not easily found through a quick internet search |  |
